# An ultra-dense integrated linkage map for hexaploid chrysanthemum enables multi-allelic QTL analysis

**DOI:** 10.1007/s00122-017-2974-5

**Published:** 2017-08-29

**Authors:** Geert van Geest, Peter M. Bourke, Roeland E. Voorrips, Agnieszka Marasek-Ciolakowska, Yanlin Liao, Aike Post, Uulke van Meeteren, Richard G. F. Visser, Chris Maliepaard, Paul Arens

**Affiliations:** 1 Plant Breeding, Wageningen University and Research, P.O. Box 386, 6708 PB Wageningen, The Netherlands; 2 Deliflor Chrysanten B.V., Korte Kruisweg 163, 2676 BS Maasdijk, The Netherlands; 3 Horticulture and Product Physiology, Department of Plant Sciences, Wageningen University, P.O. Box 16, 6700 AA Wageningen, The Netherlands; 4 Research Institute of Horticulture, Konstytucji 3 Maja 1/3, 96-100 Skierniewice, Poland

## Abstract

*****Key message***:**

**We constructed the first integrated genetic linkage map in a polysomic hexaploid. This enabled us to estimate inheritance of parental haplotypes in the offspring and detect multi-allelic QTL.**

**Abstract:**

Construction and use of linkage maps are challenging in hexaploids with polysomic inheritance. Full map integration requires calculations of recombination frequency between markers with complex segregation types. In addition, detection of QTL in hexaploids requires information on all six alleles at one locus for each individual. We describe a method that we used to construct a fully integrated linkage map for chrysanthemum (*Chrysanthemum* × *morifolium*, 2*n* = 6*x* = 54). A bi-parental F1 population of 406 individuals was genotyped with an 183,000 SNP genotyping array. The resulting linkage map consisted of 30,312 segregating SNP markers of all possible marker dosage types, representing nine chromosomal linkage groups and 107 out of 108 expected homologues. Synteny with lettuce (*Lactuca sativa*) showed local colinearity. Overall, it was high enough to number the chrysanthemum chromosomal linkage groups according to those in lettuce. We used the integrated and phased linkage map to reconstruct inheritance of parental haplotypes in the F1 population. Estimated probabilities for the parental haplotypes were used for multi-allelic QTL analyses on four traits with different underlying genetic architectures. This resulted in the identification of major QTL that were affected by multiple alleles having a differential effect on the phenotype. The presented linkage map sets a standard for future genetic mapping analyses in chrysanthemum and closely related species. Moreover, the described methods are a major step forward for linkage mapping and QTL analysis in hexaploids.

**Electronic supplementary material:**

The online version of this article (doi:10.1007/s00122-017-2974-5) contains supplementary material, which is available to authorized users.

## Introduction

A linkage map is a starting point for localization of genomic regions that are associated with agriculturally important traits. This makes it an important tool for DNA-informed breeding (Peace 2017). For polyploids, DNA-informed breeding has lagged behind compared to diploids, because genotyping co-dominant markers and linkage map construction in polyploids requires specialized methods. Such methods need to be able to handle higher dose markers. As opposed to diploids, polyploids have multiple conformations of heterozygous genotypes; on a locus with two alleles, a hexaploid can have five different heterozygous genotypes ranging from a dosage of one to a dosage of five. Together with the two homozygous conformations, this adds up to seven different dosage scores.

Many linkage maps of polyploids are constructed with single-dose (present/absent) markers using methods developed for diploids. Those kinds of maps are limited to representing only individual homologues. Integration of separate maps of homologous chromosomes is needed for transferability of results between mapping studies and mapping of traits with a complex genetic architecture. In an integrated map, all markers are located relative to each other, resulting in one representation of the positions of all mapped loci, irrespective of the phase of their alleles. This enables comparisons of linkage maps based on different populations. Map integration requires estimation of linkage between single-dose markers in repulsion or linkage between higher dose markers. Estimation of linkage of markers in repulsion is different from diploids and can only be done with very low confidence, especially in a hexaploid (Wu et al. 1992). Segregation ratios of higher dose markers are fairly complex, and calculation of recombination frequency needs specific statistical methods (Hackett et al. 1998). Because of the complicated nature of recombination frequency estimation between higher dose markers, dedicated software is required.

**Fig. 1:**
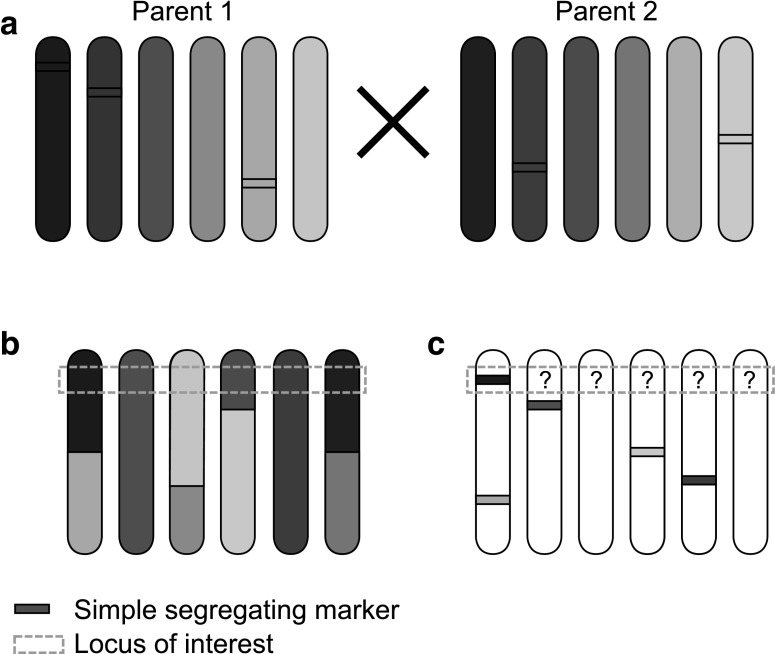
Example of a cross of two parents with each six different alleles (**a**), and an example of an F1 progeny as composed of two gametes with recombinations from both parents (**b**), and the information on the segregation based on simple 1:1 segregating markers, of which the *coloured band* indicates observed presence of a marker allele (**c**). Considering single simple segregating markers, one can only characterize absence or presence of one allele in the F1 offspring. However, the actual situation is much more complex. On that same locus there are five other alleles that all could have specific effect on the phenotype. Genotype probability models try to reconstruct the actual situation by estimating absence and presence for each allele per locus that is approaching (**b**) (colour figure online)

In an outcrossing species, the number of alleles that can affect a trait in a single individual is the same as the ploidy level (Fig. 1a). For QTL detection in an outcrossing full-sib population without any prior knowledge on the involved alleles, all twelve possible alleles that can be inherited from the parents should therefore be taken into account (Fig. 1b). With use of the positions of markers on a non-integrated linkage map of homologues, only information on the presence or absence of one out of twelve parental alleles is available (Fig. 1c). If the other eleven alleles are ignored, any QTL that does not have underlying alleles with major effect on the trait will be missed. Multi-allelic QTL mapping needs therefore information of all alleles per locus.

Most progress in linkage mapping in an outcrossing hexaploid with polysomic inheritance has been reported in sweet potato (*Ipomoea batatas*). In this species, several non-integrated maps have been published (Ukoskit and Thompson 1997; Kriegner et al. 2003; Cervantes-Flores et al. 2008; Chang et al. 2009; Monden et al. 2015; Shirasawa et al. 2017), with three publications reporting information on homologous chromosomes without actually integrating the maps. In two publications, this information is based on markers that have a dosage of two (duplex) in one parent and zero (nulliplex) in the other (2 × 0 markers), and markers with a dosage three (triplex) in one parent and zero in the other (3 × 0 markers; Ukoskit and Thompson 1997; Cervantes-Flores et al. 2008). Others have identified homologous chromosomes based on alignment to a reference genome (Shirasawa et al. 2017). Similar to sweet potato, chrysanthemum is an outcrossing hexaploid with polysomic inheritance (Van Geest et al. 2017b). Pairing at meiosis is primarily through bivalents, but multivalents do occur (Roxas et al. 1995; Chen et al. 2009). Reported methods for linkage map construction (Zhang et al. 2010, 2011a) and QTL analysis (Zhang et al. 2012a, b, 2013) for chrysanthemum have been limited to methods developed for diploids, and constructed maps are, therefore, not integrated.

In a hexaploid, an integrated linkage map is most preferably constructed by estimation of linkage with higher dose markers. Those multi-dose markers can connect homologous chromosomes within parents and between parents and can therefore be used to integrate them. For tetraploids, methods to estimate linkage between higher dose dominant markers have been developed (Hackett et al. 1998), and applied to construct integrated linkage maps (Meyer et al. 1998; Luo et al. 2001; McCallum et al. 2016). Later, these methods have been extended and applied to bi-allelic SNP markers (Hackett et al. 2013; Bourke et al. 2016, 2017). Such methods would need to be extended to hexaploids to generate integrated linkage maps with use of higher dose markers.

An integrated linkage map can be used to reconstruct inheritance of parental haplotypes to approach a representation as in the example in Fig. 1b. The two alleles of bi-allelic SNPs can be in linkage disequilibrium with multiple haplotypes, each having a different effect on the phenotype. Such haplotypes can be identified based on the configuration of neighbouring alleles. Methods for reconstruction of haplotype inheritance by estimating probabilities of identity-by-descent (IBD) in tetraploid bi-parental populations have been developed (Hackett et al. 2013; Bourke 2014; Zheng et al. 2016). Although all methods are theoretically extendible to hexaploids, the method developed by Bourke (2014) is ploidy level independent and is, therefore, directly applicable to hexaploids.

In this paper, we describe the construction of an integrated linkage map from all possible marker dosage types in hexaploid chrysanthemum. We are setting the standard for transferability of results by chromosomal linkage group numbering based on synteny with lettuce (*Lactuca sativa*) and by generating a core set of SNP markers that can be used to anchor future maps. With the integrated linkage map, we reconstruct haplotypes based on parental origins using a relatively simple procedure. We demonstrate the usefulness for QTL mapping for four traits for which information of all twelve segregating alleles was taken into account.

## Materials and methods

### Plant material and phenotyping

We analyzed the segregation of SNP markers in a bi-parental population that consisted of 406 individuals originating from a cross between DB36451 (P1) and DB39287 (P2), two daisy-type, white chrysanthemum cultivars. Phenotyping took place in the same experiment as described by van Van Geest et al. (2017a). In short, the offspring and parents were grown in three randomized blocks in each of three seasons: summer (May to July 2015), late summer (August to October 2015), and autumn (September to November 2015). A replicate consisted of a field containing 10–50 plants. Plants were grown in 12, 12 and 14 days of 18-, 21- and 21-h photoperiods for the summer, late summer and autumn, respectively. To induce flowering, they were subsequently grown in 12-h photoperiods for the plants grown in summer and late summer and in 11-h photoperiods for the plants grown in autumn. Flower colour was recorded based on visual observation. If flowers were completely white, they were scored as 0, if they were slightly pink as a 1, and pink flowers were scored as 2. Flowering time was recorded as the number of days (at short photoperiod) needed to reach commercial maturity for at least 50% of the plants grown in a single field. The number of ray florets was counted from the third flower head from the top of one flower stem for each replicate. The phenotypic scores obtained for disk floret degreening are described by Van Geest et al. (2017a). Heritability was calculated by dividing the estimated genotypic variance by phenotypic variance. Variances were estimated using an analysis of variance (ANOVA) with trial and genotype as fixed effects.

### Mitotic chromosome counting

For mitotic metaphase chromosome analysis, ±1 cm long roots were collected from DB36451 (P1) and DB39287 (P2) and incubated in eppendorf tubes in ice water for 24 h and then fixed in ethanol–acetic acid (3:1) solution for 12–24 h. Roots were stored in fixative at −20 °C until use. For chromosome preparations, the root tips were washed four times 5 min in enzyme buffer (0.01 M citric acid-sodium citrate, pH 4.8) and incubated in an enzyme mixture containing 1% (w/v) pectolyase Y23, 1% (w/v) cellulase RS at 37 °C for about 1.5 h. Squash preparations were made in a drop of 45% acetic acid and frozen in liquid nitrogen. The cover slips were removed by using a razor blade. The slides were then dehydrated in absolute ethanol, air dried and stained with 1 µg/ml 4,6-diamidino-2-phenylindole (DAPI, Sigma) in Vectashield (Vector Laboratories). Images of fluorescently stained chromosomes were acquired using a Canon digital camera attached to an Axiophot microscope with an appropriate filter and then processed using software (Axio Vision 4.2). For each genotype, the total number of chromosomes was determined for 5–10 metaphases.

### Genotyping and marker quality filtering

Genotyping was performed with a 183k Affymetrix SNP array, as described by Van Geest et al. (2017b) In short, the array was designed based on RNA-seq data of 13 cultivars, including both parents of the population. A reference transcriptome was assembled based on the reads originating from DB36451 (the female parent of the population), and reads of all 13 other cultivars were aligned against this assembly. From these alignment files SNPs were called, while retaining information from which transcript contig they originated.

Dosage scoring from array output was mainly performed as described by Van Geest et al. (2017b). Because genomic dosage was highly correlated with the number of reads per allele from our sequence data (Van Geest et al. 2017b), we estimated dosage per SNP of the parents a priori based on the sequence data, and used this information for SNP calling. This resulted in 67,916 SNP markers with expected segregation in the population based on parental dosages. Similar to the description provided by Van Geest et al. (2017b) we removed non-segregating markers, markers with >5% missing values, and skewed markers (*p* < 0.001 based on a *χ* ^2^-test assuming polysomic inheritance). Of the individuals we removed selfings and individuals with >10% missing values, resulting in 400 out of 406 individuals. We grouped identical markers together if markers had identical non-missing dosage scores for each individual. These groups of non-unique markers were represented by a single marker from that group that had the least missing values. This representative marker was used in further mapping steps with all other unique markers. After ordering, the other markers in the represented group were assigned to the same position as the representative marker.

### Linkage map construction

To calculate recombination frequency (*r*) and LOD scores of marker pairs, the method as described by Bourke et al. (2016) was modified for hexaploids, i.e., using the assumption of completely random bivalent pairing. Initially, marker dosages were converted to their most fundamental form as previously described (Bourke et al. 2016), resulting in nineteen separate marker segregation types (Online Resource 1). For all possible marker combinations (Online Resource 2), functions for pairwise estimation of *r* were then derived. In a hexaploid species, fifteen bivalent pairing scenarios are possible, in comparison to three for a tetraploid. For each combination of marker types, there are multiple phases possible depending on the conformation of the markers within one or both parents. All possible phase combinations were calculated for each marker pair (i.e., all phases having a distinct likelihood function), since the phasing of marker pairs is unknown before mapping. The recombination frequency (and associated LOD) was selected among those estimates of *r* in the range 0 ≤ *r* < 0.5 which maximized the log of the likelihood function (Hackett et al. 2013). The accuracy of recombination frequency estimation and phase assignment was checked using a small simulated hexaploid dataset generated in PedigreeSim (Voorrips and Maliepaard 2012), which showed a high degree of concordance between the true and expected results for most marker combinations. In cases where the accuracy of the estimate was lower, the LOD score reflected this (being loosely related to the inverse of the variance of the estimate). Overall, for each marker type combination (Online Resource 2) a total of 104 linkage functions were derived in Mathematica 10.0 (Wolfram Research Inc. 2015) and converted to R language (R Core Team 2014) for the linkage analysis.

To construct backbone clusters that would represent homologues, simplex × nulliplex (1 × 0) markers were clustered at a LOD score of 10. To identify chromosomal linkage groups (CLG), multi-dose markers can be used to provide bridge linkages between pairs of 1 × 0 markers, therefore associating clusters into CLG. Abundant multi-dose markers provide the most information, among those are uniparental duplex × nulliplex (2 × 0) markers (Bourke et al. 2017) or bi-parental markers, like simplex × simplex (1 × 1) markers. In our case, the use of 1 × 1 markers showed the clearest associations between 1 × 0 clusters, and these marker types were therefore used to identify CLG. Markers in clusters smaller than five markers were not used in further mapping steps. Linkage information of the bi-parental 1 × 1 markers were used to assign consensus numbering to the linkage groups between parents. After construction of this backbone clustering, all other marker types were assigned and phased to a CLG and homologue based on linkages with 1 × 0 markers with a LOD score greater than five. To complete information on all pairwise linkages, for each marker combination within a linkage group, recombination frequency and LOD were calculated with the derived functions. The markers were ordered using MDSmap (Preedy and Hackett 2016), with parameter settings as suggested by the authors: we used Haldane’s mapping function, two dimensions for the principal curves, and LOD^2^ as weights. We did not observe any notable change on the map ordering between two and three principal curve dimensions, and we therefore chose to use the simplest setting of two dimensions. After the first round, problematic markers were removed based on visual inspection of the principal curves and the difference in distance between nearest neighbouring markers as estimated from recombination frequency and the distance on the map. This difference is represented by the nearest neighbour fit (Preedy and Hackett 2016) and markers exceeding a value of four were considered problematic and thus removed. This was repeated if the next round resulted in a reduction of the total nearest neighbour fit. From the integrated map, all marker alleles were assigned to a homologue. This assignment was based on coupling linkages with 1 × 0 markers that formed the backbone clustering. If there were at least five coupling linkages with 1 × 0 markers at LOD greater than 5, alleles were assigned to a homologue. If the number of marker alleles was not equal to the number of assigned homologues, the marker was not included in the phased map.

As SNP markers were discovered from an RNA-seq-derived transcriptome assembly, each marker is associated with a transcript contig sequence. This information was used to investigate the quality of the map. Markers of the type 1 × 0 that originated from the same transcript contig should have a distance approaching 0 cM on the integrated map (assuming the contig was assembled correctly). For each homologue combination and for each linkage group, an overall deviation was quantified by calculating the root mean square error (RMSE) of these differences on the integrated map for all mapped 1 × 0 markers originating from the same transcript contig.

To enable alignment of any future linkage maps in chrysanthemum by gene sequences, markers were identified that originated from contigs representing characterized genes. For this, mapped markers were aligned to all proteins from the UniProt database from *Chrysanthemum* × *morifolium* (taxonomy ID 41568) using BLASTX (Altschul et al. 1997). Hits were filtered for alignment lengths greater than 100 and more than 95% identity. A subset of markers originating from these filtered transcript contigs spread over all linkage groups was selected to form a reference linkage map.

### Synteny with lettuce

To investigate the synteny of the integrated linkage map with lettuce (*L. sativa*), mapped transcript contigs were aligned to the mapped unigenes of lettuce as available from the Lettuce SFP Chip Project website (Truco et al. 2013) using BLAST (Altschul et al. 1997). Unique hits with an e-value smaller than 1E−100 were used to assess synteny. Chrysanthemum CLG were renumbered based on the number of alignment hits with the lettuce linkage groups.

### IBD probabilities

**Fig. 2:**
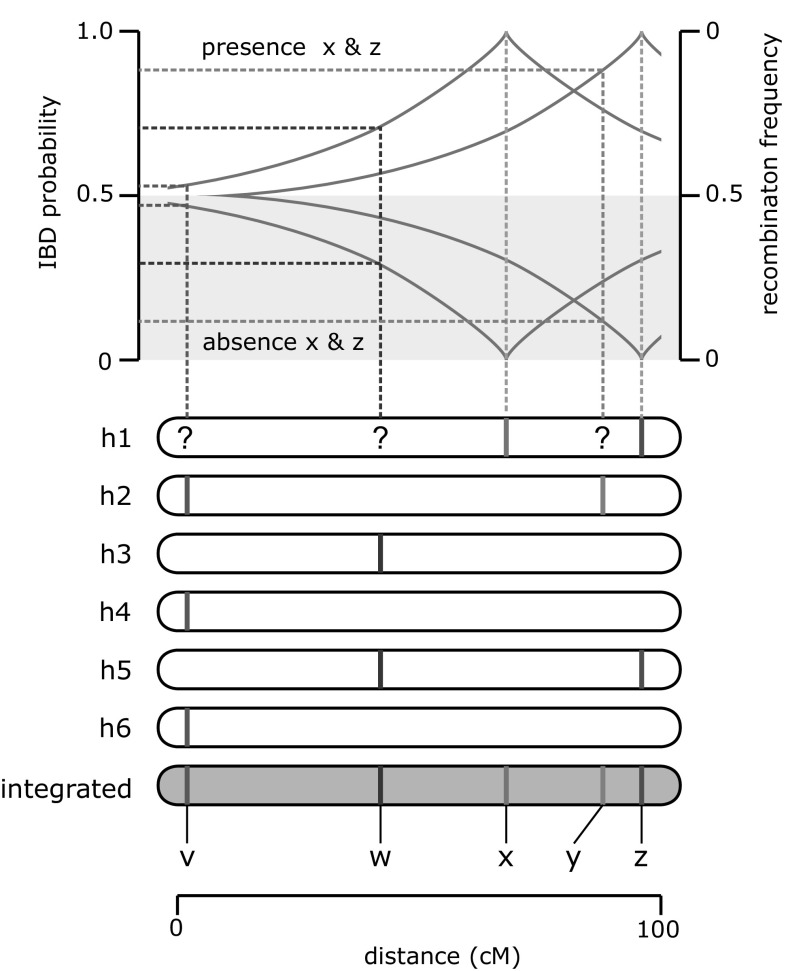
Visualisation of an example of estimating of IBD probabilities. A hypothetical integrated linkage map and the separate linkage maps of the six homologues of one parent are shown in *dark gray* and *white,* respectively. In the *upper panel* of the line graph (IBD probability >0.5), the calculation of IBD probabilities for homologue 1 (h1) are shown for marker loci v (*pink*; triplex), *w* (*purple*; duplex) and *y* (*green*; simplex) in a situation in which all alleles of marker *x* (*blue*; simplex) and *z* (*red*; duplex) are inherited. Since all alleles of loci *x* and *z* are inherited, these loci get an IBD probability of 1 for inheritance of homologue 1. If only one allele of marker *z* is inherited, this marker would be uninformative for estimating IBD probabilities, as it is not known from which homologue the single inherited allele originates (could be from h5 or h1). For marker loci *v*, *w* and *y* none of the marker alleles are present on homologue 1. It is therefore not known whether h1 is inherited at these loci. The *orange lines* depict the relationship between genetic distance and recombination frequency (*r*), as a function of map distance (Haldane’s function: $r = \frac{{1 - e^{ - 2d} }}{2}$, where *d* is distance in Morgan). Because distance between all marker combinations is known based on the integrated map, we estimate the IBD probabilities of loci *v*, *w* and *y* as 1 − *r* (in case of inheritance of all alleles of *x* and *z*), where *r* is the recombination frequency between the locus of interest and the closest informative marker (which is marker *x* in the case of *w* and *v*, and *z* in the case of *y*). The *lower panel* of the *line graph* (*shaded* in *gray*; IBD probability <0.5) depicts the situation where none of the alleles of loci *x* and *z* are inherited. Here, IBD probabilities for *v*, *w* and *y* are estimated as *r* (colour figure online)

To estimate the presence of parental haplotypes in the offspring, we calculated IBD probabilities as described by Bourke (2014). A schematic overview of the method is shown in Fig. 2. Based on the phased map and linkage information, IBD probabilities per marker locus were calculated for each member of the F1 population in two steps. The information was stored in a three-dimensional array for each chromosomal linkage group, with marker, offspring individuals and homologue on the *x*, *y*, and *z* dimensions. In the first step, only fully informative dosage scores were used to fill the IBD probability array. This means that if a progeny has inherited all alleles of a marker on specific homologues, this progeny will be assigned an IBD probability of 1 for these homologues at that marker locus. If none of the alleles are inherited, the IBD probabilities for these homologues at the locus would be 0. In general, any scores in the progeny that were larger than zero and smaller than the sum of the parental dosage scores were considered as non-informative (e.g, for a 1 × 1 marker, progeny with a dosage of 0 or a dosage of 2 were considered informative, and with a dosage of 1 non-informative). Probabilities of loci at homologues of progeny that had non-informative marker scores were given a starting probability of 0.5. In the second step, inter-marker distance was used to estimate the IBD probabilities of homologue loci with non-informative marker scores. For each marker locus at each homologue in each progeny the closest informative marker was located, and IBD probabilities were calculated based on this closest informative marker, where:

If *P*_*j*_ = 1:

$$P_{i} = 1 - r_{ij} ,$$

If *P*_*j*_ = 0:

$$P_{i} = r_{ij} ,$$

Here, *P* represents the IBD probability, *i* indicates a marker without full IBD information and *j* indicates a fully informative marker, and *r* represents recombination frequency between an informative and non-informative marker as calculated using Haldane’s mapping function from estimated distance on the integrated map. After assignment of IBD probabilities, the sum of IBD probabilities per parent was normalized to three (as there are three homologous chromosomes in a gamete). For each homologue in each F1 individual, a cubic spline was fitted over IBD probabilities versus position to calculate IBD probability interpolations over 1 cM intervals. Genotype information content (GIC) for interval *k* at homologue *h* for *n* individuals was calculated with the following formula:

$$GIC_{hk} = 1 - \frac{2}{n}\mathop \sum \limits_{i = 1}^{n} | P_{i} - { \lfloor }P_{i} {\rceil } |,$$

where

$${ \lfloor }P_{i} { \rceil } = 0, \quad 0 \le P_{i} \le 0.5,$$

$${ \lfloor }P_{i} { \rceil } = 1, \quad 0.5 < P_{i} \le 1$$

This results in a score for GIC ranging from 0 to 1, where 0 represents a locus with little information, and 1 with complete information.

### QTL mapping

QTL analysis was performed on block-corrected mean phenotypic values using an IBD probability model, as described before for tetraploids (Bourke 2014). An additive model modified from Kempthorne (1957) as suggested by Hackett et al. (2013, 2014) was modified to the hexaploid level:

$$Y = \mu + \alpha_{2} X_{2} + \alpha_{3} X_{3} + \alpha_{4} X_{4} + \alpha_{5} X_{5} + \alpha_{6} X_{6} + \alpha_{8} X_{8} + \alpha_{9} X_{9} + \alpha_{10} X_{10} + \alpha_{11} X_{11} + \alpha_{12} X_{12} ,$$

where *α*_*i*_ and *X*_*i*_ are the main effects and indicator variables for allele *i*, respectively. The parameters representing homologue 1 and homologue 7 were taken as the reference classes and were therefore omitted from the model as in all cases three alleles are inherited per parent. To calculate the significance threshold for detecting significant QTL, a thousand permutations were run with randomly permuted phenotypes (Churchill and Doerge 1994), taking the fifth percentile of the (ordered) minimum *p* values from each genome-scan analysis as an approximate significance threshold. To identify homologues affecting the trait, a simple linear model was run for each of the twelve alleles separately.

## Results

### Linkage map

**Fig. 3:**
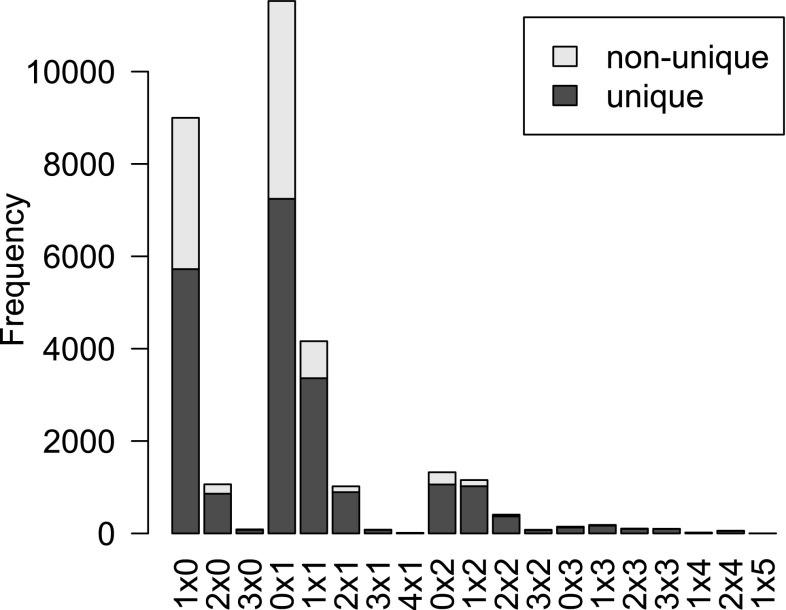
Distribution of 19 different marker types segregating in the bi-parental population. Total number of markers: 30,532, of which 21,345 were unique. The non-unique markers had duplicate dosage scores across the population. The labels on the *x*-axis represent marker segregation types such as simples × nulliplex (1 × 0), etc. (“dosage parent 1” × “dosage parent 2”)

After removal of markers that were non-segregating, had distorted segregation or had more than 5% missing values, 30,532 markers remained in the dataset. Of those, 21,345 had unique dosage scores across the progeny (Fig. 3). Because markers with identical dosage scores in each individual (non-unique markers) will map to the exact same position, they were reduced to a single, unique, marker for calculation of linkage and ordering. The others were added to the linkage map after map construction with only unique markers.

Simplex × nulliplex and nulliplex × simplex (1 × 0 and 0 × 1) markers were used to construct backbone clusters that represent homologues. This resulted in 54 clusters for P1 and 53 clusters for P2 each containing five or more markers (Online Resource 4). Chromosome counting showed that both parents had 2*n* = 54 chromosomes (Online Resource 3), the expected euploid chromosome number. Our dataset was therefore lacking 0 × 1 markers identifying one out of the 54 homologous chromosomes of P2. Identification of CLG (chromosomal linkage groups) with simplex × simplex (1 × 1) markers resulted in a network of nine CLG representing all homologue clusters of both parents. All other marker types were subsequently assigned to a CLG based on linkage with 1 × 0 and 0 × 1 markers. In total, 21,159 unique markers (99.1%) could be assigned. Markers were ordered per CLG based on recombination frequency with LOD^2^ as weights, resulting in CLG map lengths ranging from 64.5 to 95.0 cM. After ordering, the groups of non-unique markers were added to the linkage map based on the position of their unique representing marker, resulting in a linkage map containing 30,312 markers (99.3% of initial; Table 1). Of the ordered markers, the alleles of 28,638 (93.8% of initial) could be phased to an expected number of homologues based on parental dosages with at least five significant linkages to 1 × 0 markers (Table 1), resulting in a fully phased linkage map. The dosage scores of mapped markers and phased map are found in Online Resource 5 and Online Resource 6.

**Table 1:** Summary statistics of integrated linkage map

CLG	Length (cM)	Total markers	Phased markers	Contigs^a^	Rounds^b^
1	82	2595	2528	1199	2
2	77.3	3184	3110	1411	3
3	64.5	2970	2786	1269	3
4	84.2	3601	3215	1557	3
5	90.3	3498	3427	1508	3
6	91.1	3619	3533	1585	2
7	81.6	3936	3464	1621	2
8	95	3805	3499	1604	2
9	86.1	3104	3076	1338	3
Sum	752.1	30,312	28,638	13,092	–
Mean	83.6	3368	3182	1454.7	–

^a^Number of transcript contigs associated with mapped markers

^b^Number of rounds of problematic marker removal and re-ordering after the first ordering

### Synteny with lettuce and reference map

**Fig. 4:**
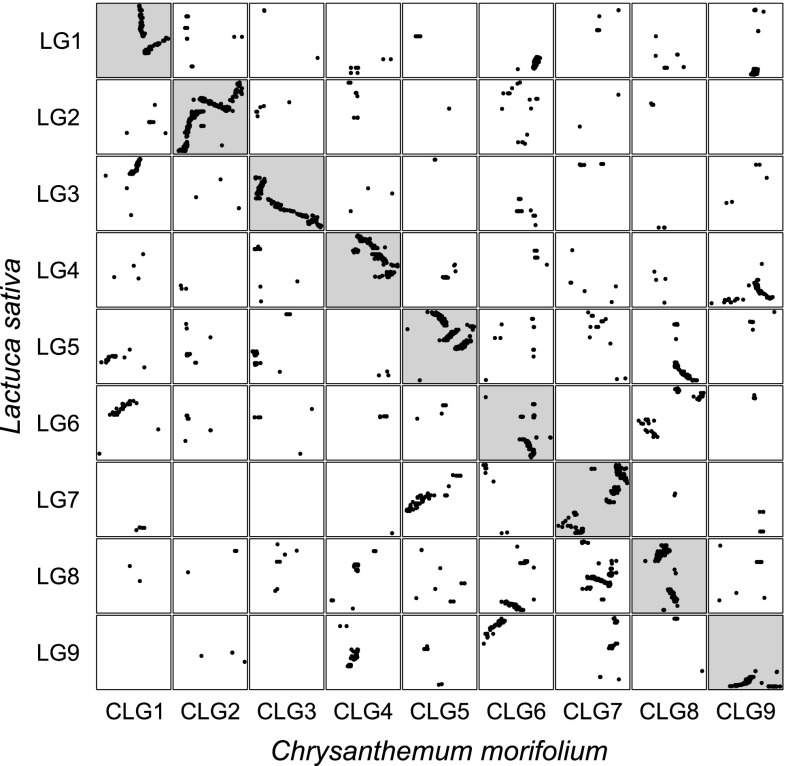
Synteny between the lettuce ultra-high density map (Truco et al. 2013) and chrysanthemum. Each dot represents a significant alignment between lettuce unigenes and chrysanthemum transcript contigs

**Fig. 5:**
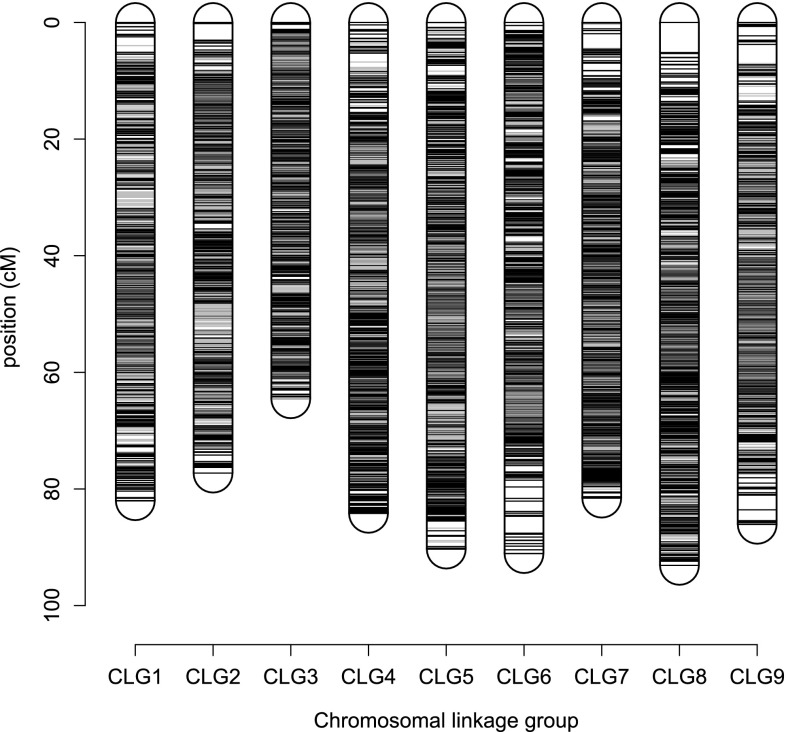
Integrated linkage map of phased markers with 1 × 0 markers (*black*), other marker types (*gray*) and CLG-defining markers (*red*) (colour figure online)

We aligned mapped transcript contigs of chrysanthemum with mapped lettuce unigenes (Fig. 4). We aligned the 13,092 mapped chrysanthemum transcript contigs to 12,841 mapped lettuce unigenes, and obtained 4757 unique hits with an e-value smaller than 1E−100. This resulted in the identification of syntenic linkage groups between lettuce and chrysanthemum. All combinations of linkage groups of chrysanthemum and lettuce with maximum number of hits were unique, except for CLG9 and CLG4 (Online Resource 7). These two CLG had both most hits with lettuce LG4. The chrysanthemum CLG9, with least hits to lettuce LG4 was renumbered based on LG9, the non-assigned linkage group from lettuce. This combination still had 126 hits, indicating partial similarity. Syntenic analysis per LG resulted in identification of large regions with linear correspondence between locations of genes, so the genomes appear to be partly co-linear at local scale. This was not clear at a larger scale, as syntenic regions were scattered across the linkage map of lettuce, which can be interpreted as that each chromosome carries major inversions and translocations. With use of this data, we based the numbering of chrysanthemum CLG on the number of significant alignments of mapped transcripts. To mark these nine chrysanthemum linkage groups, we present 92 CLG-defining SNP markers. These are evenly spread over all nine CLG and originate from 85 contigs representing genes coding for protein entries of the UniProt database (Fig. 5; Online Resource 8). This should be useful tool for future studies in chrysanthemum.

### Linkage map quality

**Fig. 6:**
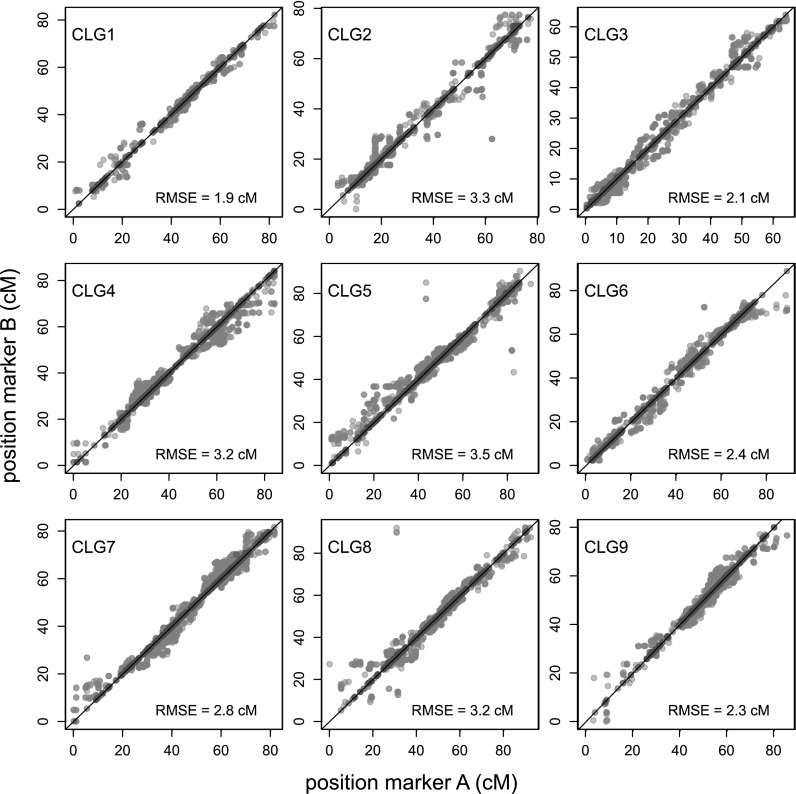
Scatterplot of marker positions of 1 × 0 markers on the integrated map that originated from the same transcript contig. Each *dot* represents a combination of markers that originated from the same transcript contig. The *red dots* indicate markers phased on the same homologue, *gray dots* on different homologues. The *black line* represents *y* = *x* (colour figure online)

We used two analyses to evaluate the quality of the linkage map. First, to investigate the concordance between estimated pairwise *r* (*r*_pairwise_) and *r* based on map distance (*r*_map_), these two estimators of *r* were plotted against each other (Online Resource 9). With high LOD scores, these two estimators were in concordance with each other over a wide range of *r* (from 0 to 0.3). Second, to evaluate the position of nearby 1 × 0 markers in coupling and repulsion, we aligned the position on the integrated map of 1 × 0 markers that originated from the same transcript contig from the RNA-seq assembly. The positions of markers that originated from the same contig and had the same phase (6937 markers in total) aligned nearly perfectly (Fig. 6), indicating low error rates. The position of markers phased on different homologues (8352 markers in total) was more spread. The residual mean squared error (RMSE) was calculated for each linkage group (Fig. 6) and each combination of homologues from the same linkage group (Online Resource 10). RMSE was generally below 5 cM, with some outliers, on CLG 2, 5 and 8. These outliers were caused by one, two, and two markers respectively.

### IBD probabilities

The presence of each of the twelve segregating haplotypes per locus was estimated in all progeny individuals at 1 cM map intervals, which was expressed in IBD probabilities. In the middle of the CLG, the IBD probabilities could be estimated with high confidence (Online Resource 4). If there were no markers in large parts of one homologue, the IBD probabilities could still be close to 0 or 1, because information from the five other homologues can complement the missing information. Even if no markers were mapped on the entire homologue, e.g., homologue 12 from P2 on linkage group 4, IBD probabilities were complemented with information from the other five homologues. Genotype information content was lower towards telomeres, because in those regions markers were often missing in a large range in at least two homologues and informative markers were present on only one side.

### QTL mapping

The population was phenotyped for four different traits: flower colour, flowering time, disk floret degreening and number of ray florets. All four traits had a moderately high heritability ranging from 0.68 to 0.72 (Online Resource 11). The phenotypes were fitted against the IBD probabilities at 1 cM intervals with a main effects model.

**Fig. 7:**
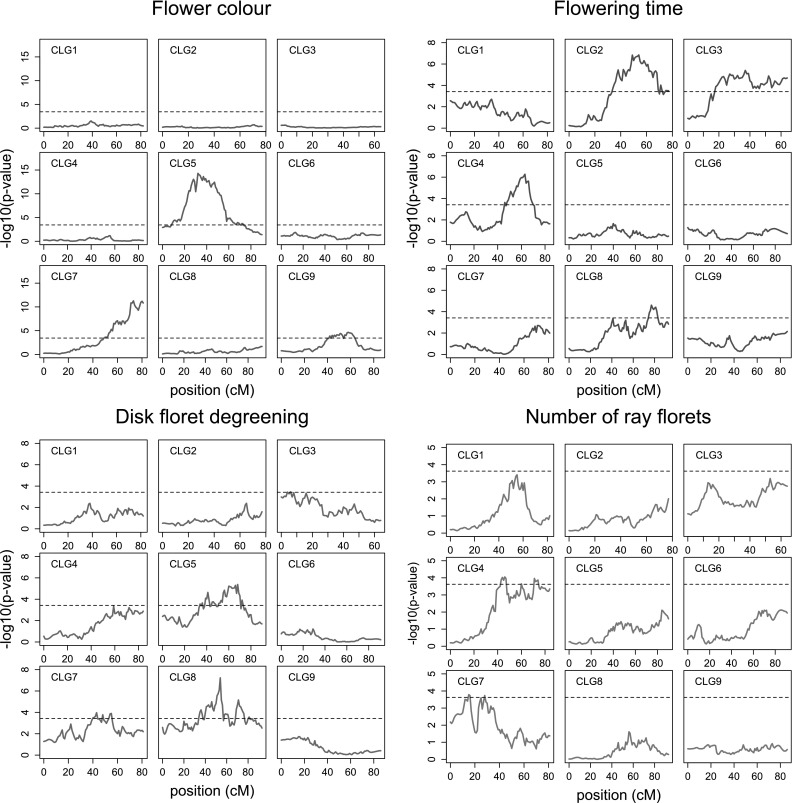
QTL analysis of flower colour (*purple*), flowering time (*red*), disk floret degreening (*green*) and number of ray florets (*blue*). Significance thresholds based on 1000 permutations (see “Materials and methods”) are indicated with the *dashed line* (colour figure online)

**Fig. 8:**
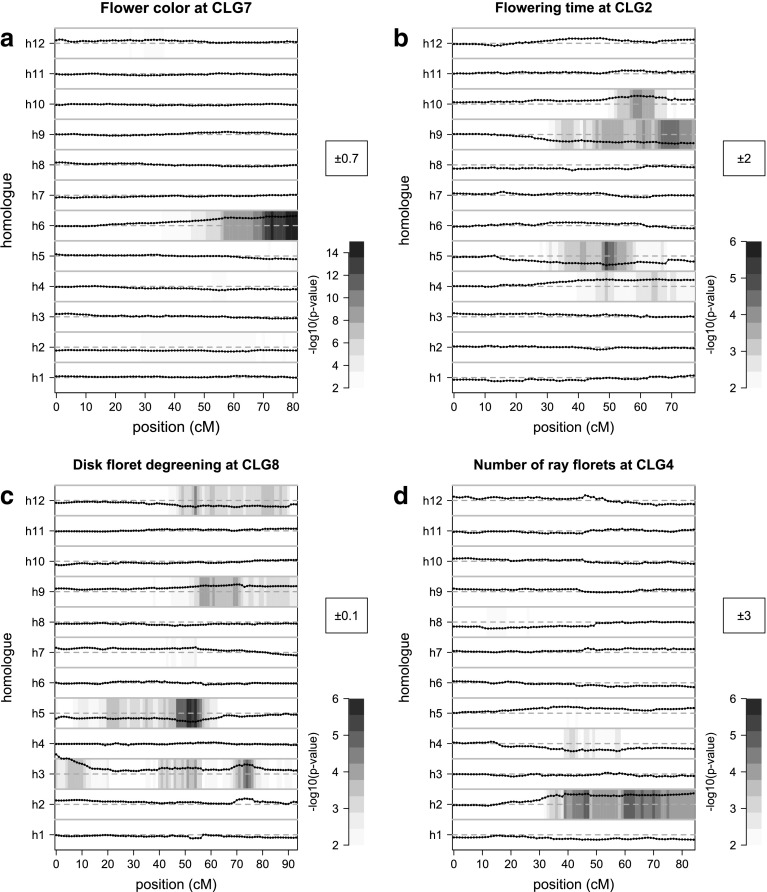
Analysis per homologue for four QTL: flower colour at CLG7 (**a**), flowering time at CLG2 (**b**), disk floret degreening at CLG8 (**c**), and number of ray florets at CLG4 (**d**). The *p* value for testing the significance of the explained variation of IBD probabilities of a single allele versus phenotype ($Y_{\text{G}} = \mu_{\text{C}} + \alpha_{i} X_{i}$) is shown as a heatmap. The estimated effect of full absence or presence of an allele on the phenotypic value is shown in the *black points*. The plot limits of the effect is shown in the *black box*, meaning that the *black points* can range within the negative and positive value between the boundaries between homologous indicated by *gray lines*. The *dotted gray lines* represent an effect of zero

Two regions were highly significantly associated with flower colour, at CLG5 and 7, and one region at CLG9 was slightly associated (Fig. 7). The highly significant loci were both simplex QTL (Fig. 8a; Online Resource 12; Online Resource 13a, b). Analysis of variance of the interaction between the associated alleles showed a highly significant (*p* < 1E−16) interaction, indicating that both alleles need to be present to get a pink flower colour. Together, the two 1 × 0 markers that were most closely linked to each of the QTL explained 47.8% of the variation, indicating that the trait is mainly inherited by two major alleles segregating from two loci from each of the two parents. There is a minor QTL on CLG9, but some genotypic variation is still to be explained by undetected QTL.

For flowering time, we found three clear QTLs on CLG2, 3 and 4 and one minor QTL at CLG8 (Fig. 7; Online Resource 12; Online Resource 13c–e). For the simplex QTL on CLG4, presence or absence of the allele at homologue 11 had a major effect on the trait (Online Resource 12; Online Resource 13d). In both loci on CLG2 and 4 presence of different alleles had a positive effect, a negative effect or no significant effect on the phenotype (Fig. 8b; Online Resource 12; Online Resource 13c). Therefore, at least three alleles underlie the QTLs.

For disk floret degreening, three QTL located on CLG5, 7 and 8 were detected (Fig. 7). For all three QTL, multiple alleles played a role (Online Resource 13f, g; Fig. 8c). The QTL on CLG8 explained most phenotypic variation, and presence of the allele on homologue 5 had the strongest effect on the mean value of disk floret degreening.

For number of ray florets, two minor QTL were found on CLG4 and 7. The QTL on CLG7 was affected by one allele from the maternal parent (Online Resource 12; Online Resource 13h). The QTL on CLG4 was affected by alleles that originated from only the maternal parent with opposite effects from different homologues (Online Resource 12; Fig. 8d).

## Discussion

In this paper, we report the first integrated linkage map in a hexaploid species with polysomic inheritance. We were able to assign multi-dose markers to their parental homologues. With this phasing information, we could reconstruct inheritance of haplotype alleles in the bi-parental population and perform QTL analyses. We provide major steps to overcome a number of limitations to linkage map construction based on SNP markers in hexaploids, including full map integration and phasing.

### An integrated and phased linkage map

The ultra-dense integrated map contained markers of all 19 possible types in a hexaploid (Online Resource 1 and Online Resource 2). With our approach, we first defined backbone marker clusters that represented homologues based on linkages between simplex × nulliplex (1 × 0) markers. With 1 × 1 markers that contained information about homologous chromosomes, we created networks of 1 × 0 linkage groups that represented CLG (chromosomal linkage groups). Subsequently the other marker types were assigned to these backbone clusters. Because we first defined backbone marker clusters based on 1 × 0 markers, definition of homologues relied on presence of 1 × 0 markers. Lack of 1 × 0 markers on a homologue is possible if there is a high degree of inbreeding, the population is a selfing, or if there is selection for a phenotype for which alleles have an additive effect. However, our experience in polyploid mapping to date has shown that low-dosage markers tend to be the most abundant marker type (Bourke et al. 2016, 2017; Van Geest et al. 2017b). Nevertheless, we were not able to define one homologue on linkage group 4, even though all others each had 175 SNP markers or more. Too few markers, therefore, seem not very likely. A reason could be that some combinations with alleles on this homologue might have been lethal, and 1 × 0 markers on this homologue might therefore have had highly distorted segregation. These markers would have been filtered out prior to linkage mapping.

The MDSmap algorithm (Preedy and Hackett 2016) has proved particularly useful for the weighted ordering of our large number of diverse marker types. As the LOD for linkage varies for the same values of *r* for different marker type combinations and phases, a weighted ordering algorithm was required. The most frequently used algorithm for weighted ordering is based on a weighted linear regression (WLR) algorithm as deployed in JoinMap (Van Ooijen 2006). However, current processor speed of desktop computers limits the use of this algorithm to approximately a hundred markers per linkage group. Because of this restriction, Bourke et al. (2016) used the WLR algorithm to construct homologue maps separately, which were later integrated. In a subsequent mapping study in tetraploid rose, the MDSmap algorithm was used to construct an integrated map containing over 25k SNPs, without the need for binning or the separation of homologue maps before integration (Bourke et al. 2017). The MDSmap algorithm also forms the core of the map-ordering module within TetraploidSNPMap software (Hackett et al. 2017), although its release as a separate R package opens up the possibility of high density mapping at any conceivable ploidy level given pairwise recombination frequency information. With the MDSmap algorithm, we were able to order all markers from a CLG in one run (ranging from 1721 to 2404 markers per CLG), resulting directly in an integrated map. This number of markers would previously have been completely intractable using a WLR. With the new algorithm, such maps can be produced on an average desktop computer within hours. As the ordering step is time and resource efficient, running multiple rounds of mapping with removal of problematic markers is much more feasible.

Linkage map quality was assessed based on two analyses: on the concordance between *r*_pairwise_ and *r*_map_, and on the relative position between 1 × 0 markers originating from the same contig from our transcriptome assembly. According to the comparison of *r*_pairwise_ and *r*_map_, the two estimators were in concordance if *r*_pairwise_ could be estimated with high confidence (high LOD). Therefore, there is little discrepancy between the distance and ordering of different combinations of markers on the linkage map and their initial estimation of *r*. The second analysis resulted in information on the quality of local integration of homologous chromosomes. This is based on the assumption that recombinations are essentially absent within a transcript contig. Therefore, we would expect that markers originating from the same contig have a distance very close to 0 cM. From the position of markers originating from the same contigs, a difference from zero can be calculated, and with that a measure for error; we used the RMSE. The RMSE of 1 × 0 markers mapped on the same homologue was generally very low indicating both a high-quality assembly of transcripts containing mapped markers, and high-quality local ordering at the level of the homologue. The RMSE of 1 × 0 markers phased on different homologues was higher. Of all marker type combinations, the estimation of genetic distance between 1 × 0 markers in coupling is most accurate. Estimation of distance between 1 × 0 markers in repulsion relies on higher dose markers, because high-confidence estimation of recombination frequency in repulsion of 1 × 0 markers was not possible with our population size of 406. The positions of 1 × 0 markers that are on different homologues relative to each other are therefore estimated with lower certainty than if they were in coupling phase. However, errors in estimating distance between these 1 × 0 markers in repulsion were in general lower than 5 cM. If serious ordering issues occurred, a much higher value would be anticipated. Nevertheless, there were four homologue combinations with RMSE values higher than 10 cM. Only one or two markers per CLG caused these high values. As these markers were not associated with any notable stress on the map, it is likely that these markers were actually from different loci in the genome, and the contigs they originated may be the result of a chimeric contig assembly from two very similar transcripts originating from the same chromosome.

Earlier linkage maps of chrysanthemum are based on RAPD, ISSR, AFLP (Zhang et al. 2010) and SRAP markers (Zhang et al. 2011a). A disadvantage of these types of molecular markers is that they are difficult to transfer, and different linkage maps therefore cannot be integrated. SNP markers are sequence based, and executing single SNP assays like KASP™ or TaqMan™ are commonly applied laboratory procedures. They can therefore be flawlessly transferred between laboratories. To set a standard for chrysanthemum, we present the sequences of a set of 92 well-distributed SNP markers originating from conserved coding sequences that can be used as a core set to align future linkage maps to each of the chromosomal linkage groups presented here.

### Estimating IBD probabilities

We used a relatively simple approach to estimate IBD probabilities for absence or presence of parental haplotypes in our segregating population (Bourke 2014). The method only uses information of dosage scores if they are fully informative. This means that in case of a 1 × 1 marker for example, a dosage of 0 and a dosage of 2 in the progeny is fully informative (while assuming absence of double reduction), because it represents inheritance of, respectively, none of the associated homologues or both. A dosage of 1 is not fully informative as it is not known from which parental homologue the allele originated. Therefore, higher dose markers carry relatively few informative dosage scores. A consequence of our method is that it is only accurate if markers with a large fraction of informative dosage scores are equally distributed over the homologues. In our data, parts of homologues were sometimes poorly endowed with informative markers. This did not turn out to be problematic if at that position all other five homologues for that parent carried enough information. More sophisticated methods have shown that higher dose markers add more information to the estimation of IBD probabilities (Hackett et al. 2013; Zheng et al. 2016). Such methods could result in more accurate IBD estimates, but an adequate marker distribution over all homologues is key to all methods.

The accuracy of genetic analysis based on IBD probabilities relies on the quality of the integrated map. If the estimation of distance between markers with alleles on different homologues is poor, estimation of IBD probabilities of alleles on the presumed same locus will be wrong, and will, therefore, provide a poor representation. However, the RMSE of the marker positions on the integrated map was generally well below 5 cM. This would not have a large effect on the estimation of IBD probabilities, because according to Haldane’s mapping function a distance of 5 cM corresponds to a recombination frequency of 0.047, resulting in a relatively low error of 4.7% on the estimation of IBD probabilities.

### QTL mapping

With the integrated map and IBD probabilities, we were able to perform a multi-allelic QTL analysis. In a polyploid, this type of analysis has large advantages over the use of methods that are developed for diploids, because QTL that are regulated by multiple different alleles can be detected and their genetic architecture investigated (Hackett et al. 2014). In a polyploid, more than two alleles can underlie a QTL. This means that the QTL genotype does not only have a dosage, but can also be multi-allelic (i.e., not only different conformations of the alleles A and B, but also combinations of, e.g., A, B, C, D, E and F are possible within a locus). To investigate the genetic architecture and with that the occurrence of multi-allelic QTL, we performed a QTL analysis that makes use of using IBD probabilities for four traits with different underlying genetic architecture.

The major loci associated with flower colour were bi-allelic. Together, they explained a large part (47.8%) of the phenotypic variation and were affected by one allele for each of the two loci. The two loci clearly showed an interaction, suggesting that presence of both alleles is needed for pink colouration. In chrysanthemum, pink colouration is caused by anthocyanin accumulation (Stickland 1972). The interaction between alleles could be caused by the requirement of two enzyme variants needed for the production or regulation of production of anthocyanin, or two gene copies that are required for the same limiting step, needing the additive effect of both to become visible.

Several QTLs associated with flowering time, disk floret degreening and number of ray florets were multi-allelic. These QTLs had underlying alleles with a positive effect, a negative effect and no significant effect on the phenotype, indicating presence of at least three alleles. The exact number of unique alleles that affect the phenotype is difficult to determine. Two haplotypes that have the same effect on the phenotype could have the same underlying polymorphism affecting the phenotype, which would make them the same alleles. On the other hand, they could contain different causative polymorphisms that have a similar effect on the phenotype. Based on our data, it is not possible to uniquely identify such alleles, because our analysis is based on genetic linkage, and the causative alleles cannot be identified.

Compared to flower colour, the genetic architecture for flowering time was more complex. The QTL at CLG4 was bi-allelic, meaning that presence of one allele affected the trait, whereas the other eleven alleles did not significantly affect the phenotype. However, in two other major QTL on CLG2 and 3 multiple alleles were involved. Other studies on the inheritance of flowering time in chrysanthemum also suggested involvement of multiple loci (Zhang et al. 2011b, 2013). Flowering time in short day plants is mainly the result of an interaction between growth rate and signal transduction of environmental cues like day-length and temperature. As these cues are strictly controlled in a greenhouse, the role of the environment would be expected to be relatively small. This is supported by the relatively high heritability (0.70), which was also found earlier (De Jong 1984). However, genetic regulation of signal transduction and growth rate is likely complex and it is therefore not surprising that multiple loci are involved.

Disk floret degreening is an important determinant of postharvest performance of chrysanthemum after long storage (Van Geest et al. 2017a). Three multi-allelic QTL were identified. These QTL explained a relatively small fraction of the phenotypic variation. Disk floret degreening is a physiologically complex trait; in the investigated population it is related to carbohydrate content of the disk floret at harvest (Van Geest et al. 2017a). Many sub-traits could affect carbohydrate content, including genotypic variation related to photosynthetic rate and source–sink relationships. Furthermore, it was shown that carbohydrate content is not the only factor affecting degreening (Van Geest et al. 2016). It is, therefore, not surprising that we did not find major QTL for disk floret degreening. Dissecting the trait further by phenotyping for sub-traits such as carbohydrate content, or by backcrossing progeny harboring specific trait characteristics might help to further identify specific loci underlying this complex trait.

The number of ray florets had the highest heritability of the investigated traits (0.72), but least variation could be explained by detected QTL. Asteraceae plants carry composite flower heads that are comprised of multiple florets. Those florets can be categorized into disk florets and ray florets. The number of ray florets is affected by the number of florets on a capitulum and organ identity of those florets. Regulation of floret identity is generally inherited through one or two major loci in Asteraceae (Gillies et al. 2002). It is, therefore, quite unexpected we did not find any major QTL associated with the trait. As both parents were of the single flower type, it is possible that both lacked allelic variation in the major genes, and we only found variation in more complexly regulated minor allelic effects.

In the QTL analyses, possible interactions between alleles were not taken into account. An alternative model as described by Hackett et al. (2014) that uses all possible genotype classes as parameters would enable detection of interactions. However, the method we used to estimate IBD probabilities is not able to estimate probabilities for these genotype classes directly. More importantly, in a tetraploid, there are 36 possible genotype classes $$\left( {\left( {\begin{array}{*{20}c} 4 \\ 2 \\ \end{array} } \right) \times \left( {\begin{array}{*{20}c} 4 \\ 2 \\ \end{array} } \right)} \right)$$, leading to a model with 36 parameters that is already prone to over-fitting. In a hexaploid this would be 400 genotype classes $$\left( {\left( {\begin{array}{*{20}c} 6 \\ 3 \\ \end{array} } \right) \times \left( {\begin{array}{*{20}c} 6 \\ 3 \\ \end{array} } \right)} \right)$$, leading to 400 parameters; over-fitting would definitely become an issue.

Our results show that hexaploidy in chrysanthemum complicates QTL analysis because multiple alleles with a differential effect can underlie an associated locus. With the integrated map and IBD probabilities we were able to identify inheritance of parental haplotypes in the progeny, enabling us to identify effects of specific alleles that affected the phenotype. We indeed found clear examples in which different alleles from the same locus and parent affected the trait negatively or positively. With these findings we show that polyploids with polysomic inheritance can harbor much more diversity on a single locus compared to a diploid, and this is very important to take into account during QTL detection and breeding.

## Conclusions

The methods described in this paper enable construction of integrated linkage maps in hexaploids with polysomic inheritance. Our presented methods can be used for future projects that aim to construct integrated linkage maps and perform multi-allelic QTL analyses in hexaploids. Success of such projects depends on several features of the investigated organism and the obtained dataset. First, it depends on the predominance of random bivalent pairing at meiosis. Second, sufficient and evenly distributed 1 × 0 markers are required that can define each homologous chromosome. Last, higher dose co-dominant markers (both uni-parental and bi-parental, e.g., 1 × 1, 2 × 0 and 3 × 0) with alleles on each homologous linkage group are needed that provide information to integrate homologous linkage groups into chromosomal linkage groups. With the resulting integrated linkage maps, it is possible to perform QTL analysis that takes all possible alleles into account at the same locus. This has major impact on the possibilities for localization of genomic loci and their genetic architecture associated with traits in chrysanthemum, but also for other agriculturally important hexaploid species such as sweet potato, kiwi and persimmon.

### **Data availability statement**

All data generated or analyzed during this study are included in this published article and its supplementary information files.

### **Author contribution statement**

GG designed and executed the phenotyping and genotyping experiments, performed the analysis and wrote the manuscript. AP, UM, RGFV, CM, REV and PA participated in coordination. REV designed the methodology for SNP dosage scoring. PMB designed the methodology for construction of linkage maps and QTL analysis together with CM, REV, GG, YL and PA. AMC developed and executed the karyotyping. All authors participated in drafting the manuscript.

## Electronic supplementary material

Below is the link to the electronic supplementary material.

Online Resource 1. Dosage conversions for each marker type. (XLSX 14 kb)

Online Resource 2. Network representing all linkage functions. The dots represent a marker type as “dosage parent 1” × “dosage parent 2”. The edges represent each possible function to calculate linkage between the two marker types. Within each function, multiple phase combinations are possible. Black lines represent unique functions, gray lines represent that were interchangeable with a representing unique function. (PDF 40 kb)

Online Resource 3. DAPI-stained metaphase chromosomes of the parents of the F1, DB36451 (A) and DB39287 (B). (PDF 199 kb)

Online Resource 4. GIC and marker distribution for each linkage group. GIC is depicted in the bars running from yellow (GIC = 0.2 to blue (GIC = 1). Vertical lines represent markers, in which 1 × 0 markers are depicted in black, and other marker types in gray. (PDF 311 kb)

Online Resource 5. Marker dosage scores of parents and offspring (XLSX 36692 kb)

Online Resource 6. Phased linkage map. Homologues are represented as h1 to h12, where h1-6 originate from P1, and h7-12 for P2. The number one in a column of a homologue indicates that the allele originated from that homologue. (XLSX 2128 kb)

Online Resource 7. Number of significant alignment hits (E-value < 1E − 100) for each combination of chrysanthemum CLG with lettuce LG. (XLSX 13 kb)

Online Resource 8. Position, gene and sequence of linkage group defining markers. (XLSX 19 kb)

Online Resource 9. Scatterplots between pairwise estimation of *r* (*r*_pairwise_) and *r* based on distance on the ordered linkage map (*r*_map_). Colour of the dots is based on LOD score of *r*_pairwise_. LOD scores greater than 50 are depicted in yellow. (PDF 4337 kb)

Online Resource 10. Heatmap of RMSE of all combinations of 1 × 0 markers originating from the same contig, for each homologue combination. (PDF 21 kb)

Online Resource 11. Summary of phenotypes (XLSX 8 kb)

Online Resource 12. Summary statistics of the different QTL. (XLSX 10 kb)

Online Resource 13. QTL analysis per homologue. Legend as in Fig. 8. (PDF 139 kb)
