# Supplementary figures and images for: An ultra-dense integrated linkage map for hexaploid chrysanthemum enables multi-allelic QTL analysis
Source: Theor Appl Genet. 2017 Aug 29;130(12):2527–41. doi: 10.1007/s00122-017-2974-5 (PMC5668331; doi:10.1007/s00122-017-2974-5)

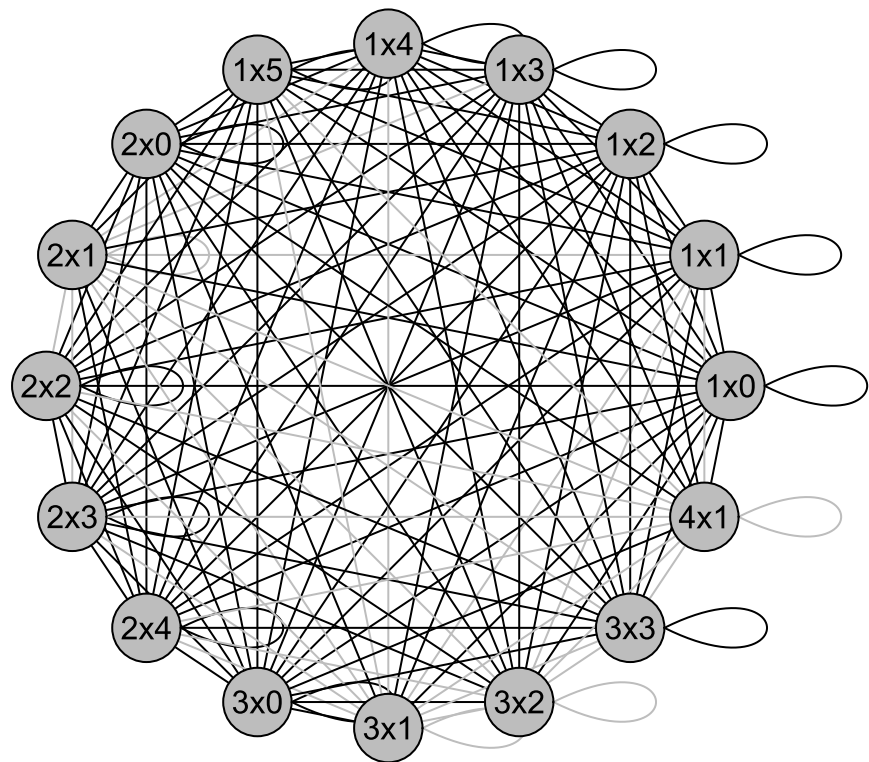

Supplement: Supplementary file 2 — Online Resource 2. Network representing all linkage functions. The dots represent a marker type as “dosage parent 1” × “dosage parent 2”. The edges represent each possible function to calculate linkage between the two marker types. Within each function, multiple phase combinations are possible. Black lines represent unique functions, gray lines represent that were interchangeable with a representing unique function. (PDF 40 kb) [file 122_2017_2974_MOESM2_ESM.pdf]

A

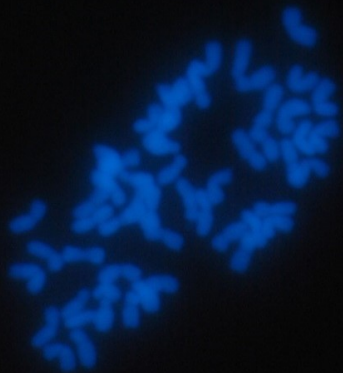

B

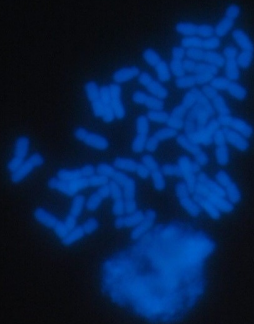

Supplement: Supplementary file 3 — Online Resource 3. DAPI-stained metaphase chromosomes of the parents of the F1, DB36451 (A) and DB39287 (B). (PDF 199 kb) [file 122_2017_2974_MOESM3_ESM.pdf]

# CLG1

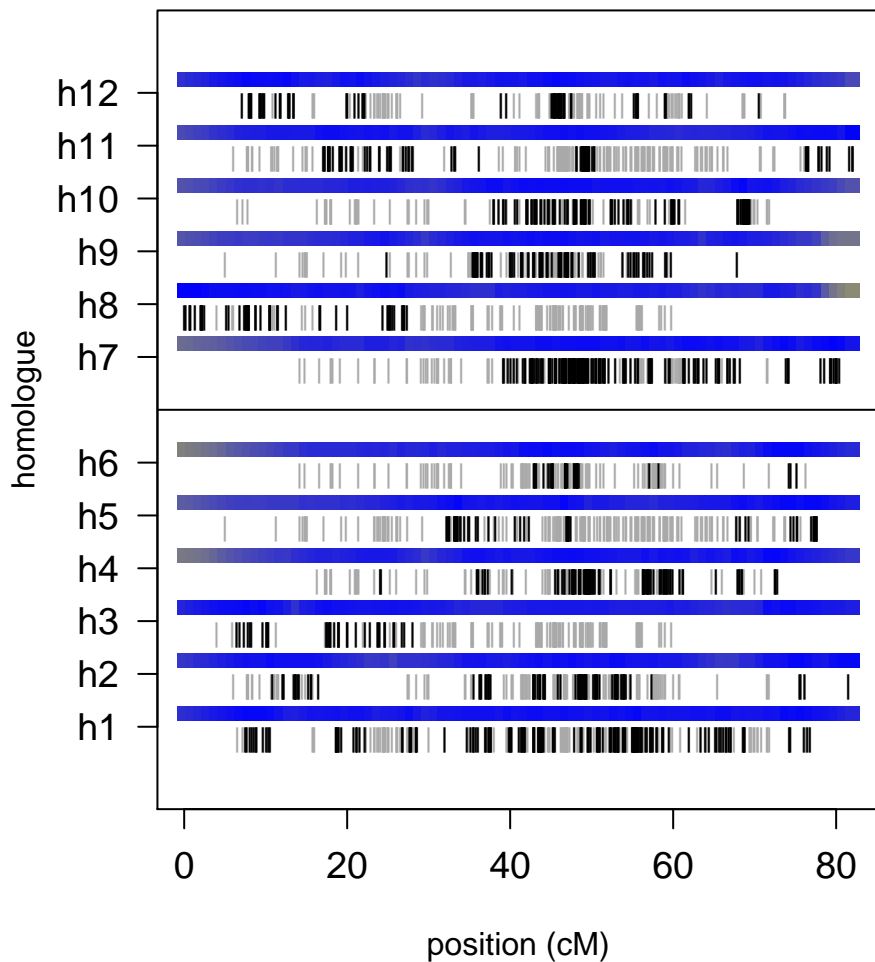

# CLG2

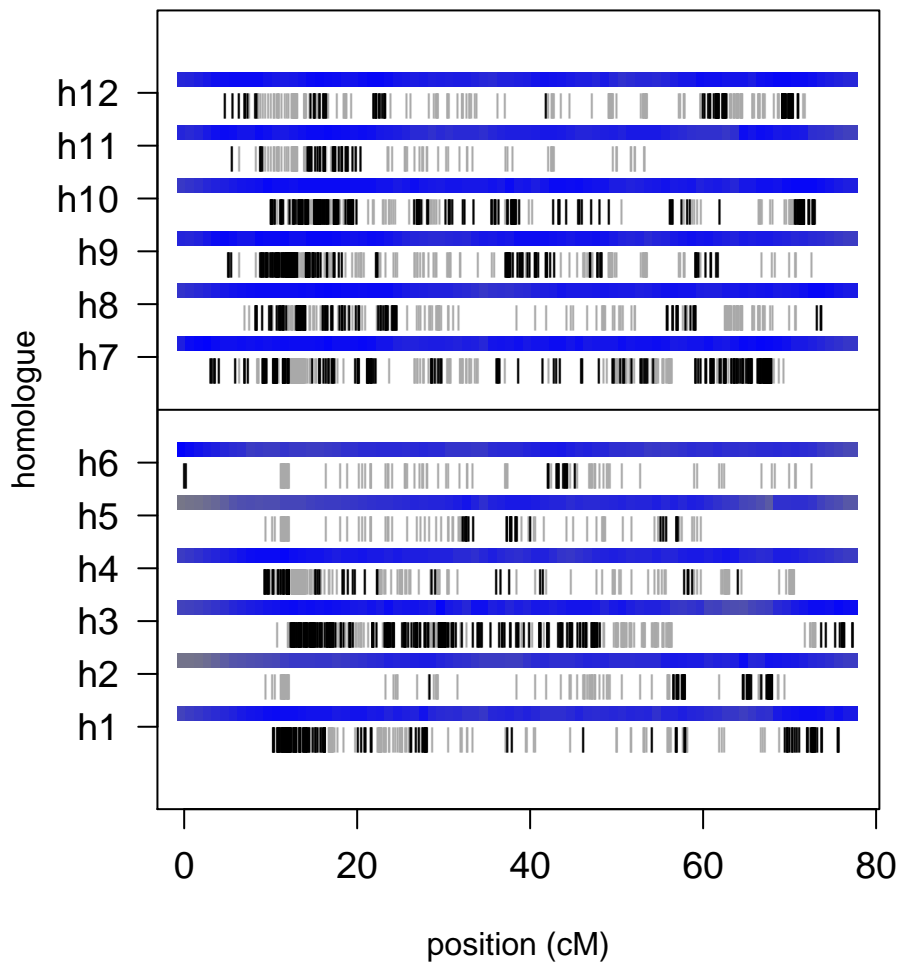

# CLG3

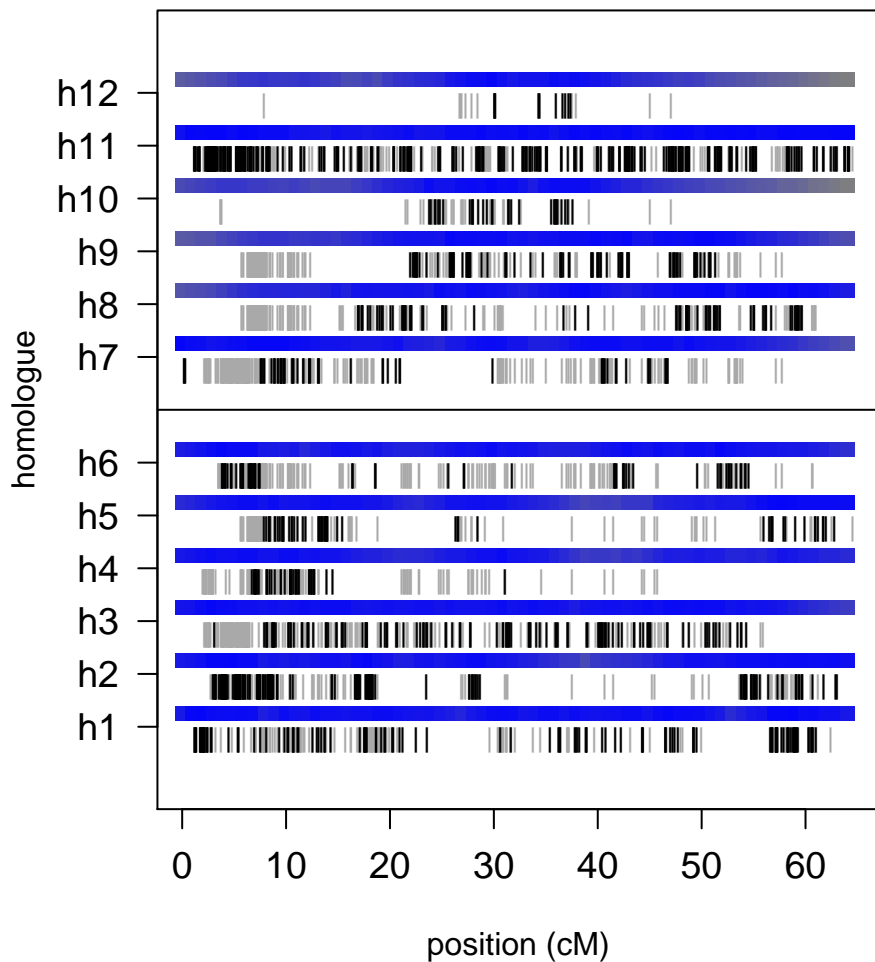

# CLG4

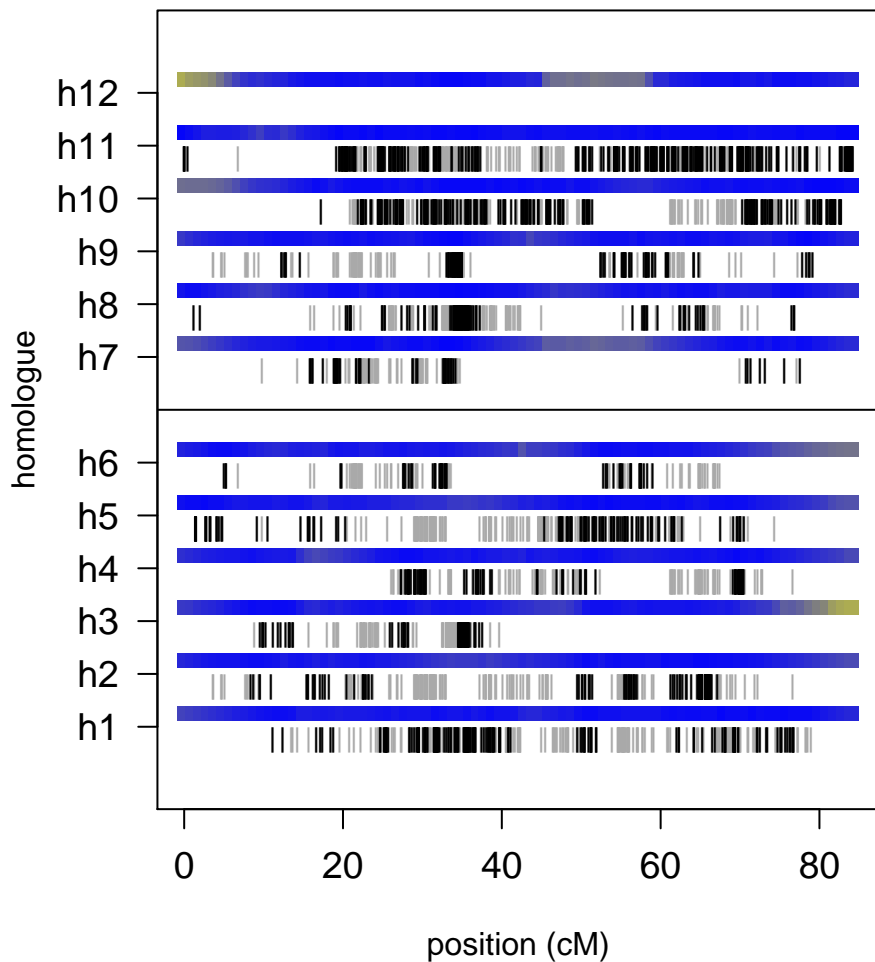

# CLG5

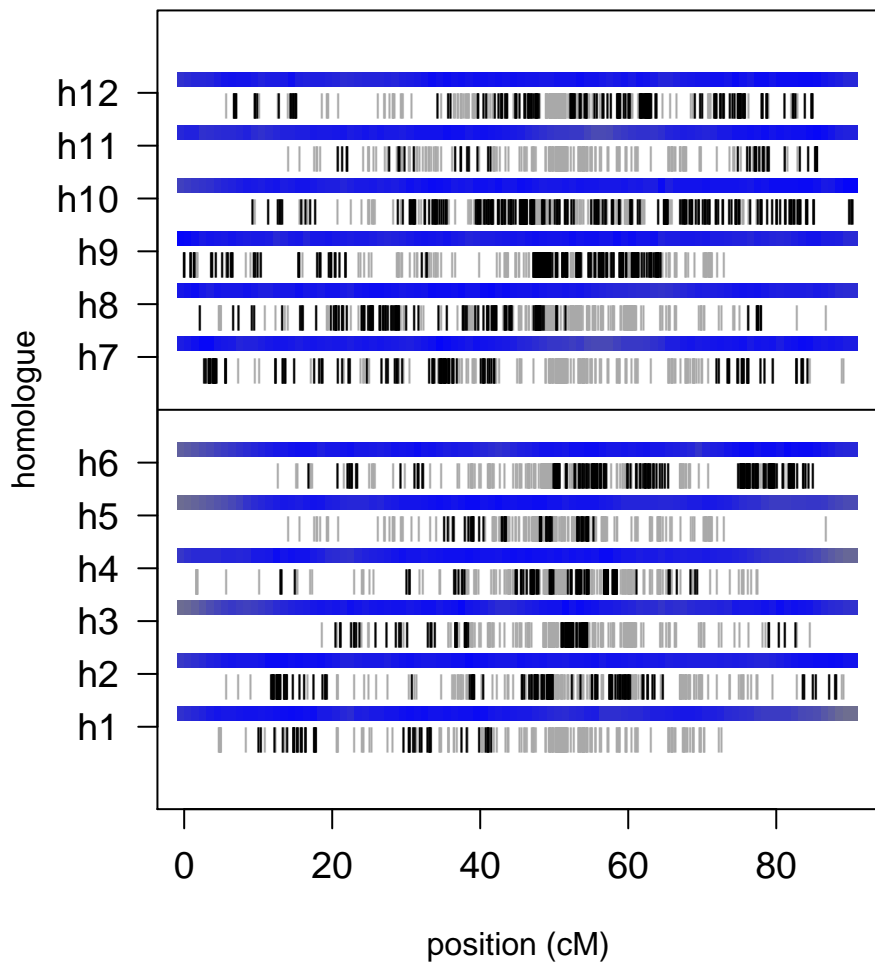

# CLG6

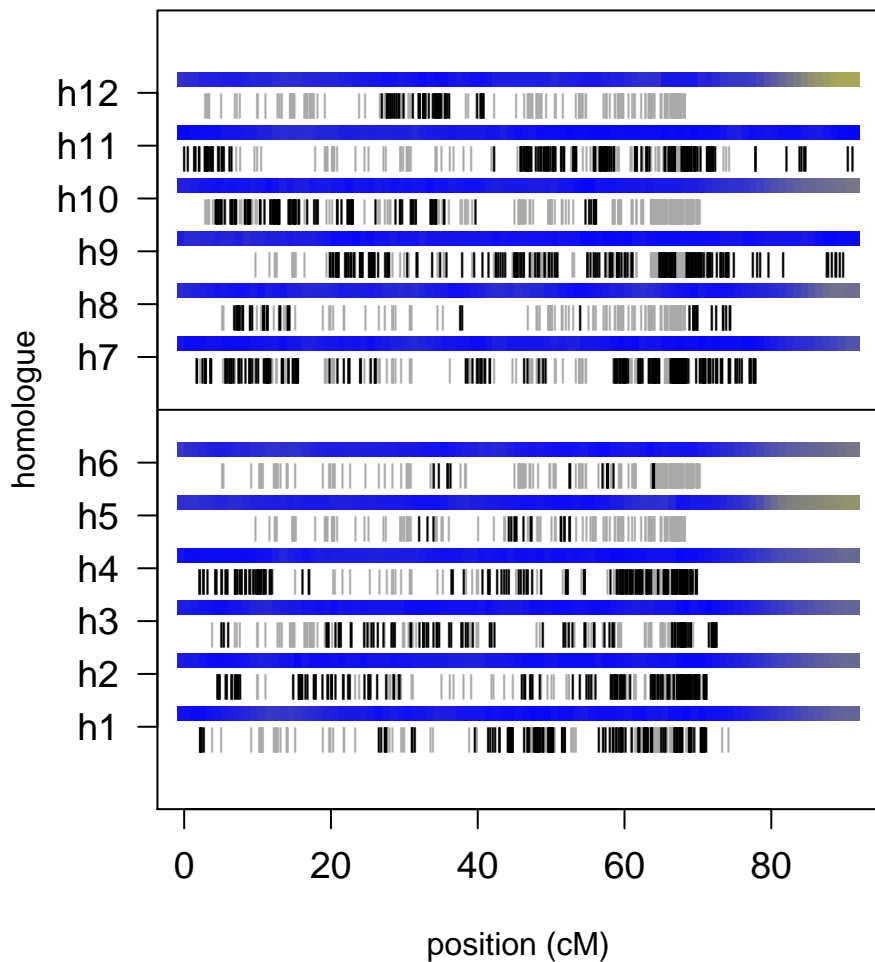

# CLG7

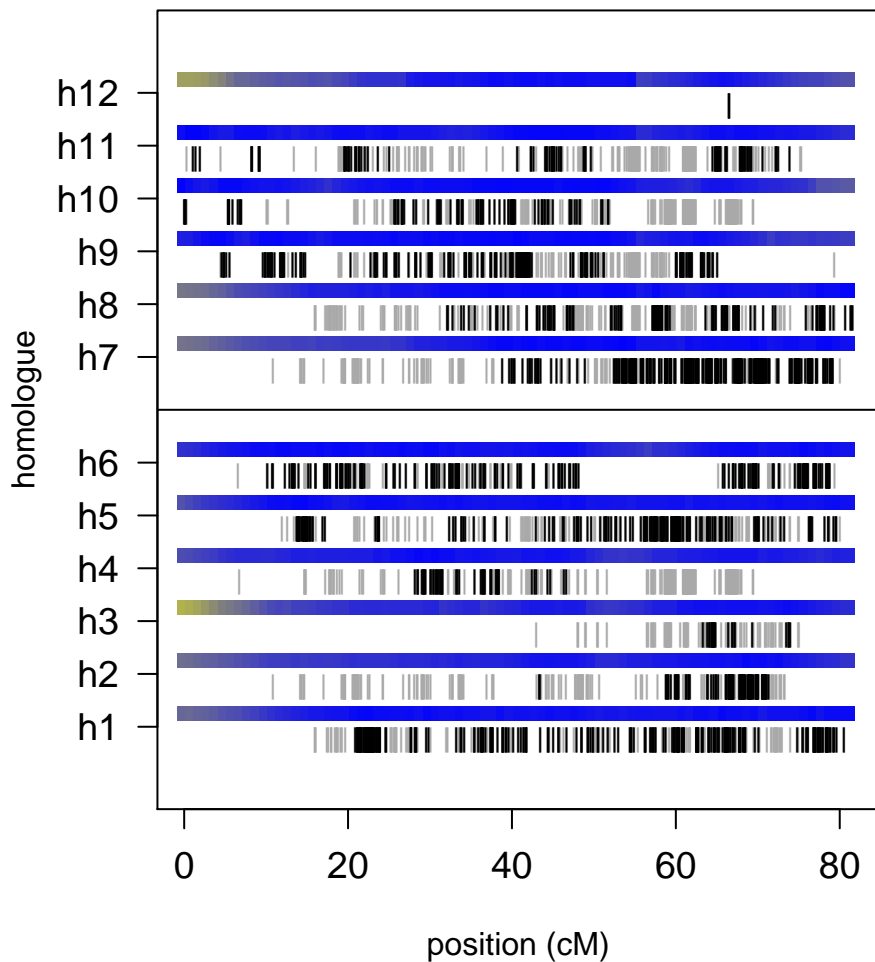

# CLG8

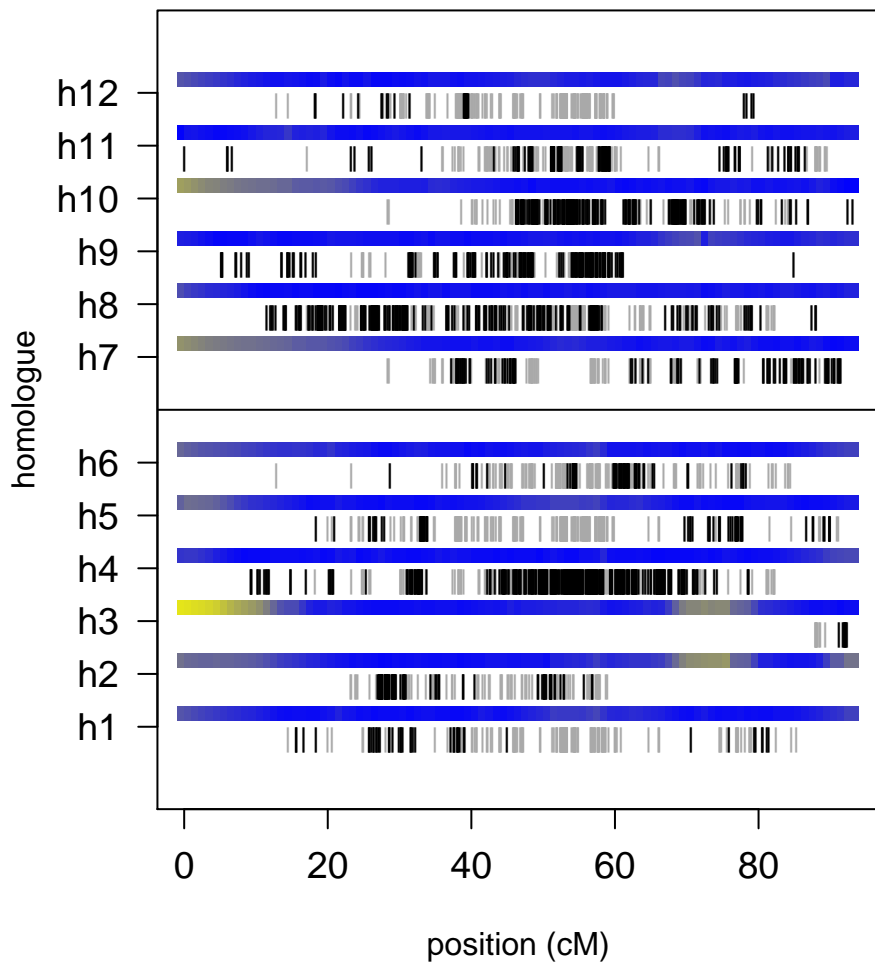

# CLG9

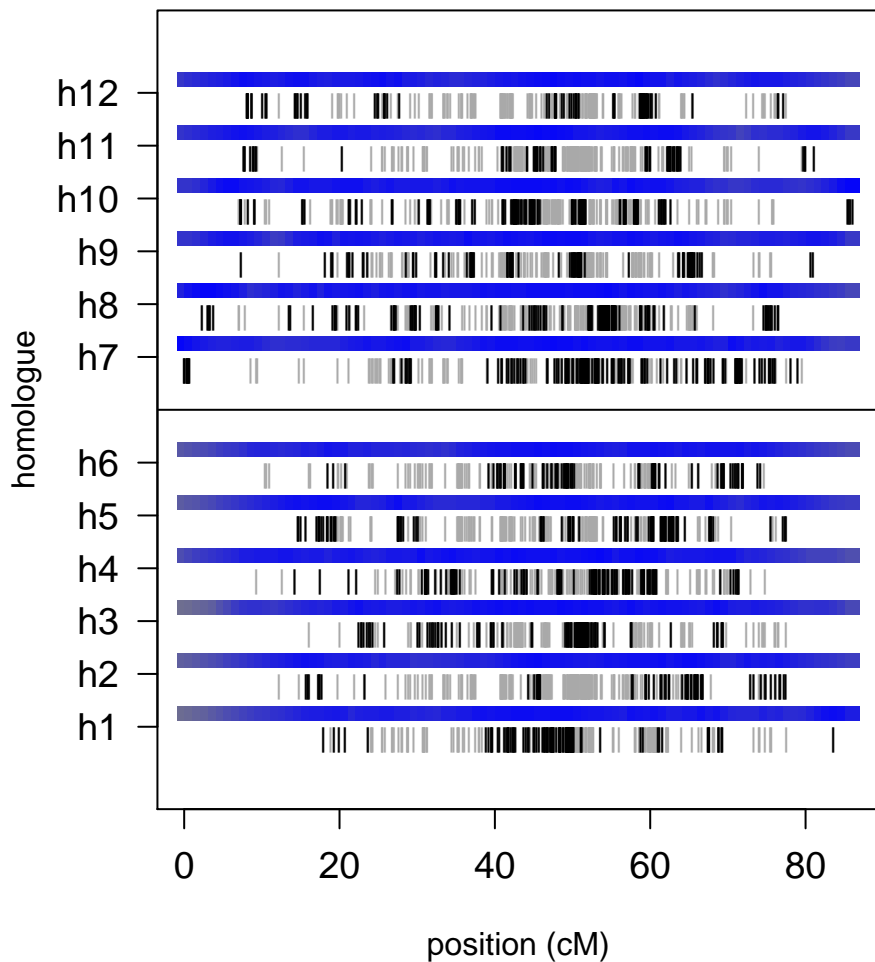

Supplement: Supplementary file 4 — Online Resource 4. GIC and marker distribution for each linkage group. GIC is depicted in the bars running from yellow (GIC = 0.2 to blue (GIC = 1). Vertical lines represent markers, in which 1 × 0 markers are depicted in black, and other marker types in gray. (PDF 311 kb) [file 122_2017_2974_MOESM4_ESM.pdf]

$r_{\text{map}}$

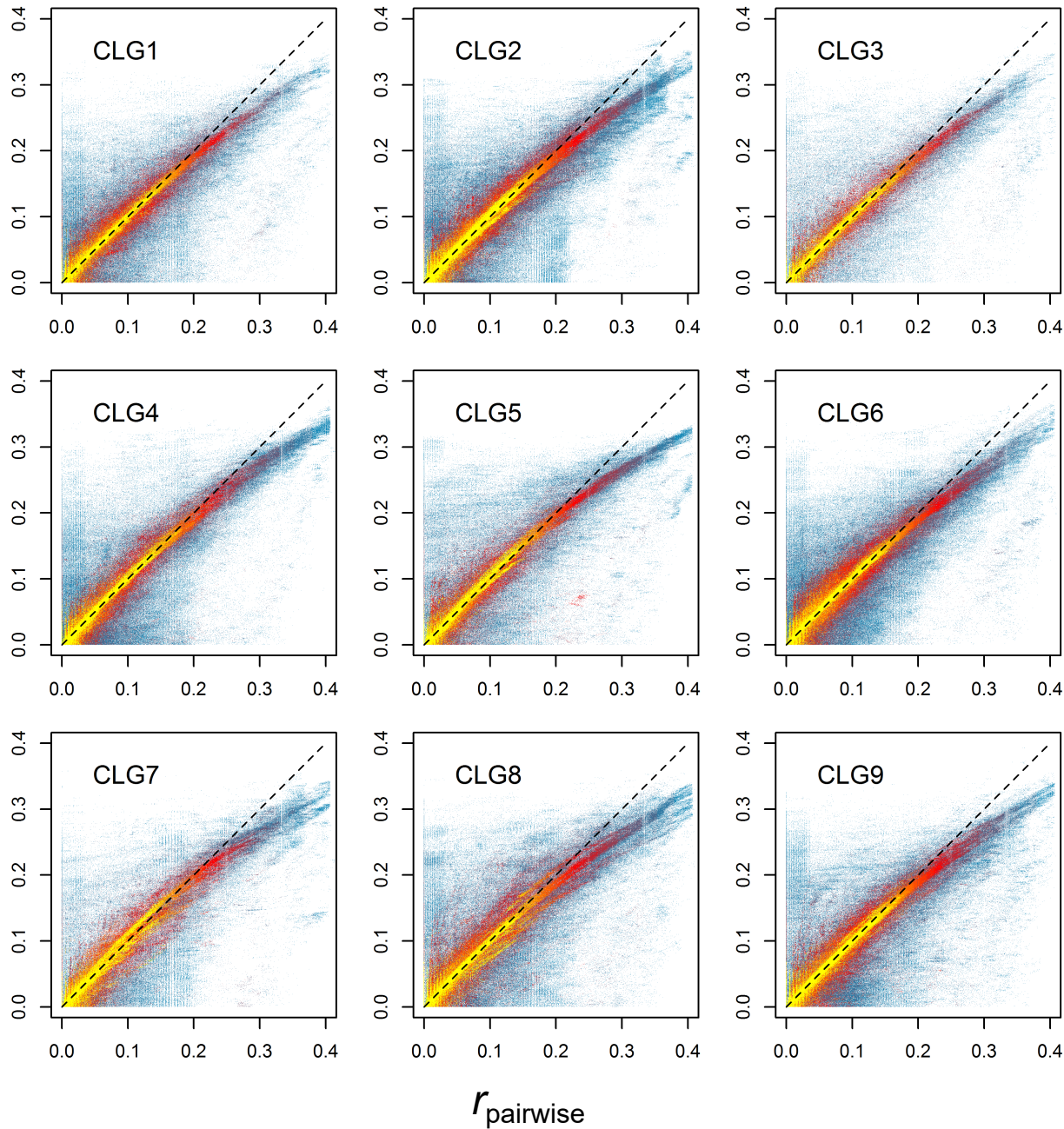

Supplement: Supplementary file 9 — Online Resource 9. Scatterplots between pairwise estimation of r (r pairwise) and r based on distance on the ordered linkage map (r map). Colour of the dots is based on LOD score of r pairwise. LOD scores greater than 50 are depicted in yellow. (PDF 4337 kb) [file 122_2017_2974_MOESM9_ESM.pdf]

CLG1

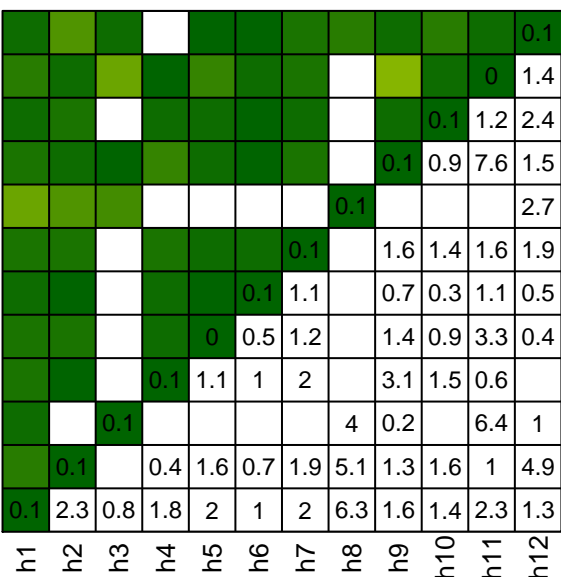

CLG2

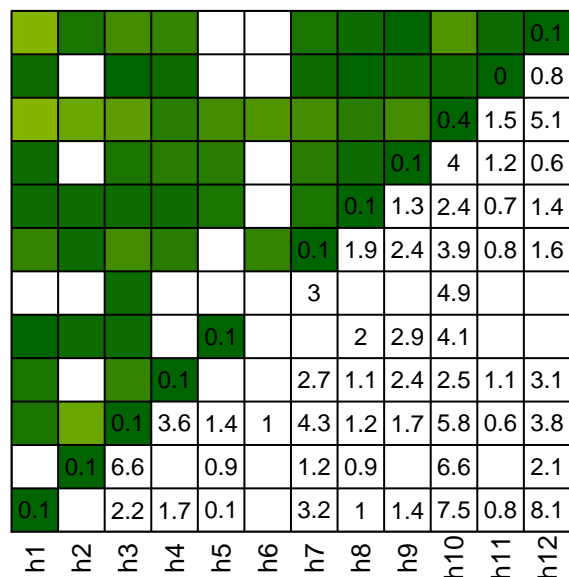

CLG3

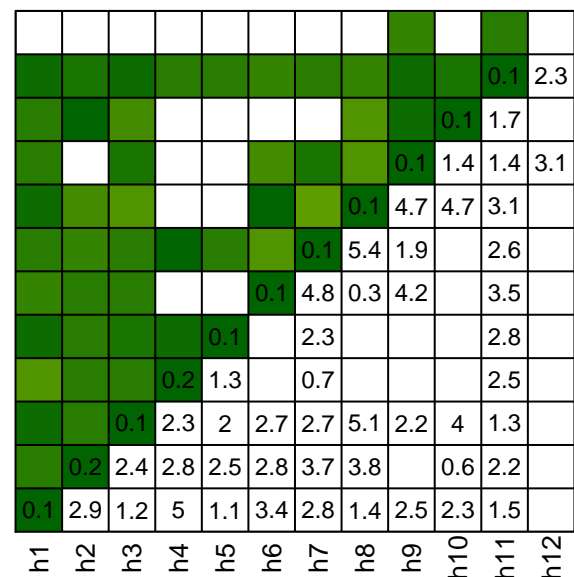

CLG4

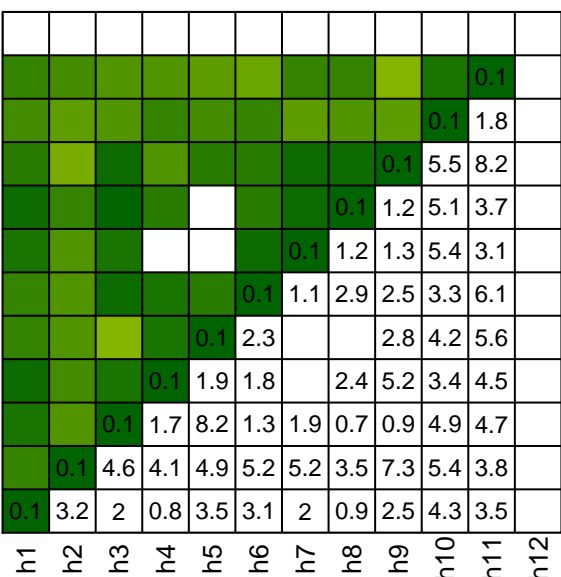

CLG5

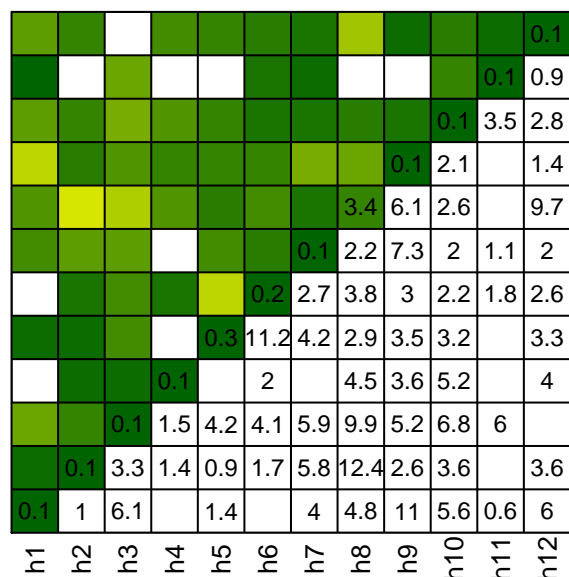

CLG6

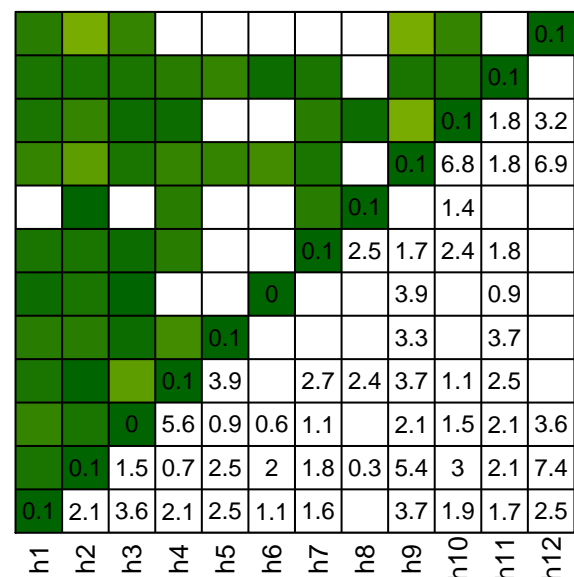

CLG7

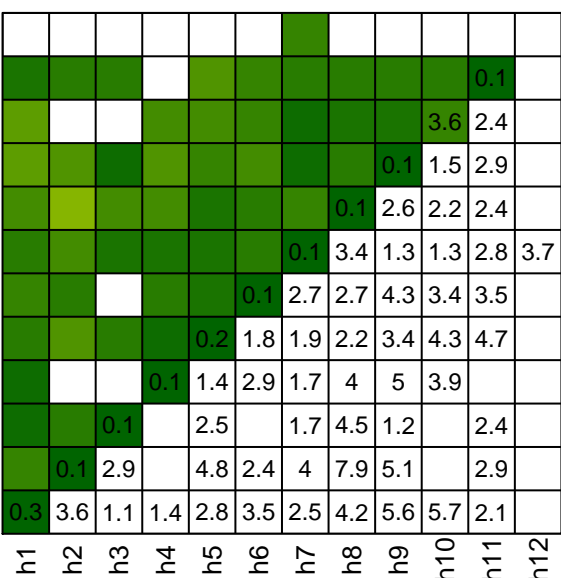

CLG8

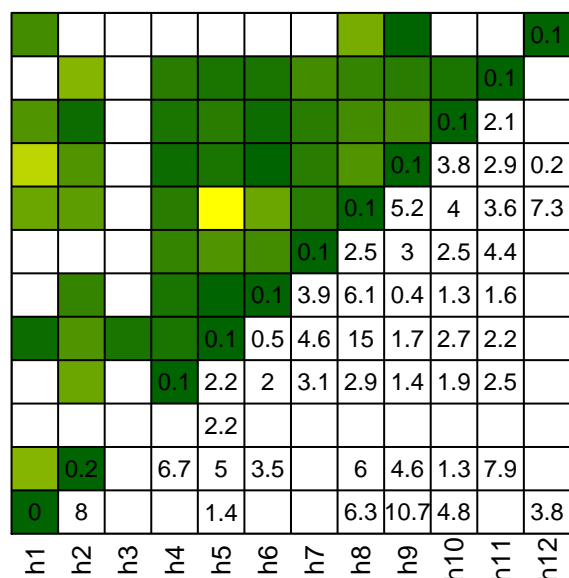

CLG9

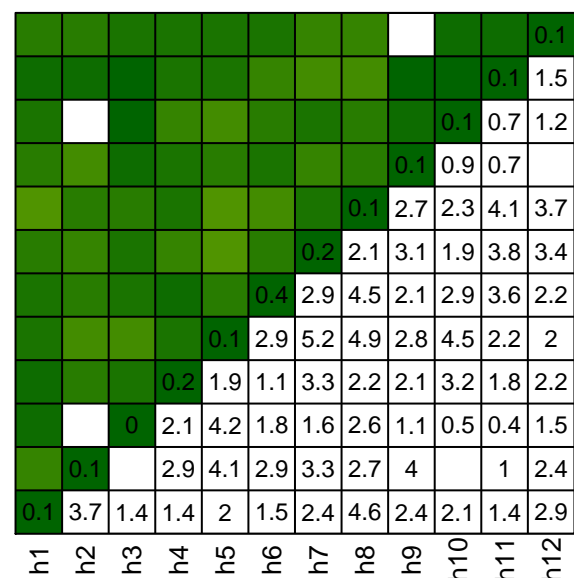

Supplement: Supplementary file 10 — Online Resource 10. Heatmap of RMSE of all combinations of 1 × 0 markers originating from the same contig, for each homologue combination. (PDF 21 kb) [file 122_2017_2974_MOESM10_ESM.pdf]
