# Supplementary material for: An ultra-dense integrated linkage map for hexaploid chrysanthemum enables multi-allelic QTL analysis
Source: Theor Appl Genet. 2017 Aug 29;130(12):2527–41. doi: 10.1007/s00122-017-2974-5 (PMC5668331; doi:10.1007/s00122-017-2974-5)
Supplement: Supplementary file 13 — Online Resource 13. QTL analysis per homologue. Legend as in Fig. 8. (PDF 139 kb) [file 122_2017_2974_MOESM13_ESM.pdf]

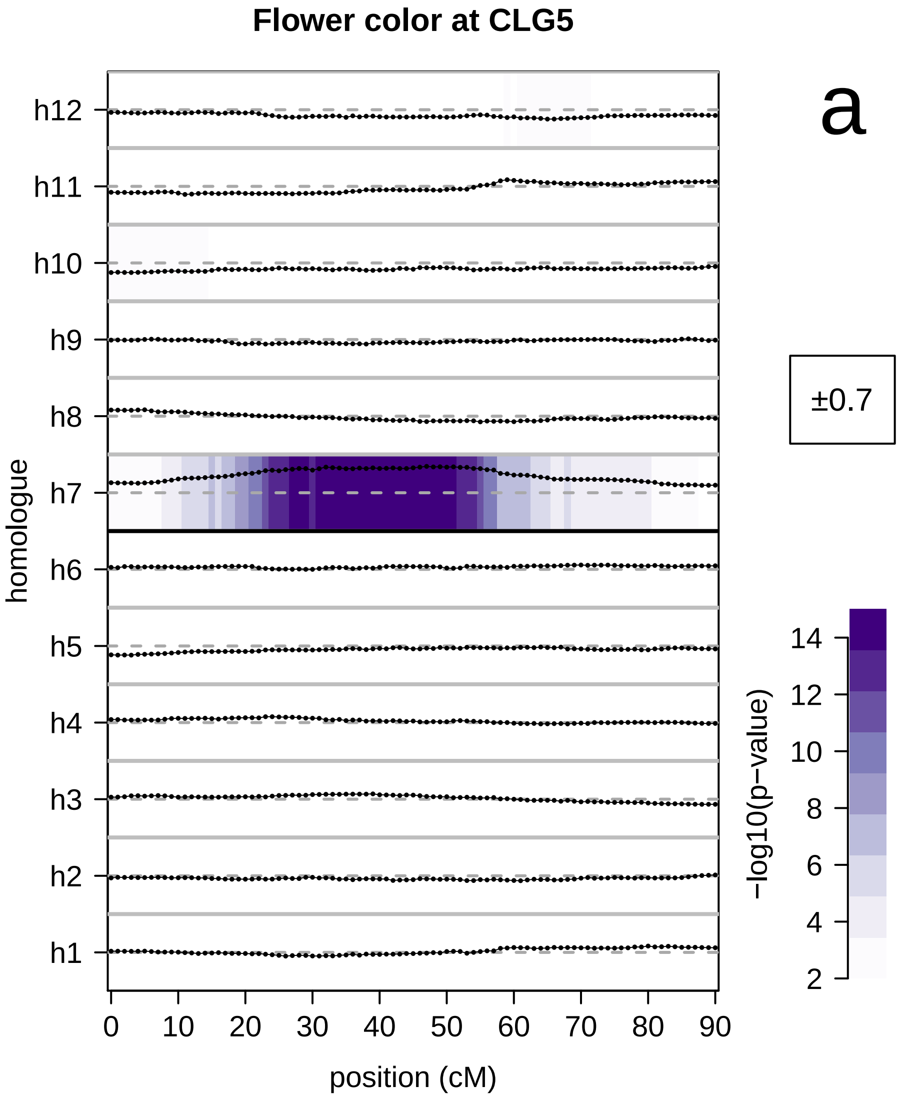

Flower color at CLG5
a
h12
h11
h10
h9
h8
h7
h6
h5
h4
h3
h2
h1
homologue
0
10
20
30
40
50
60
70
80
90
position (cM)
±0.7
−log10(p−value)
14
12
10
8
6
4
2

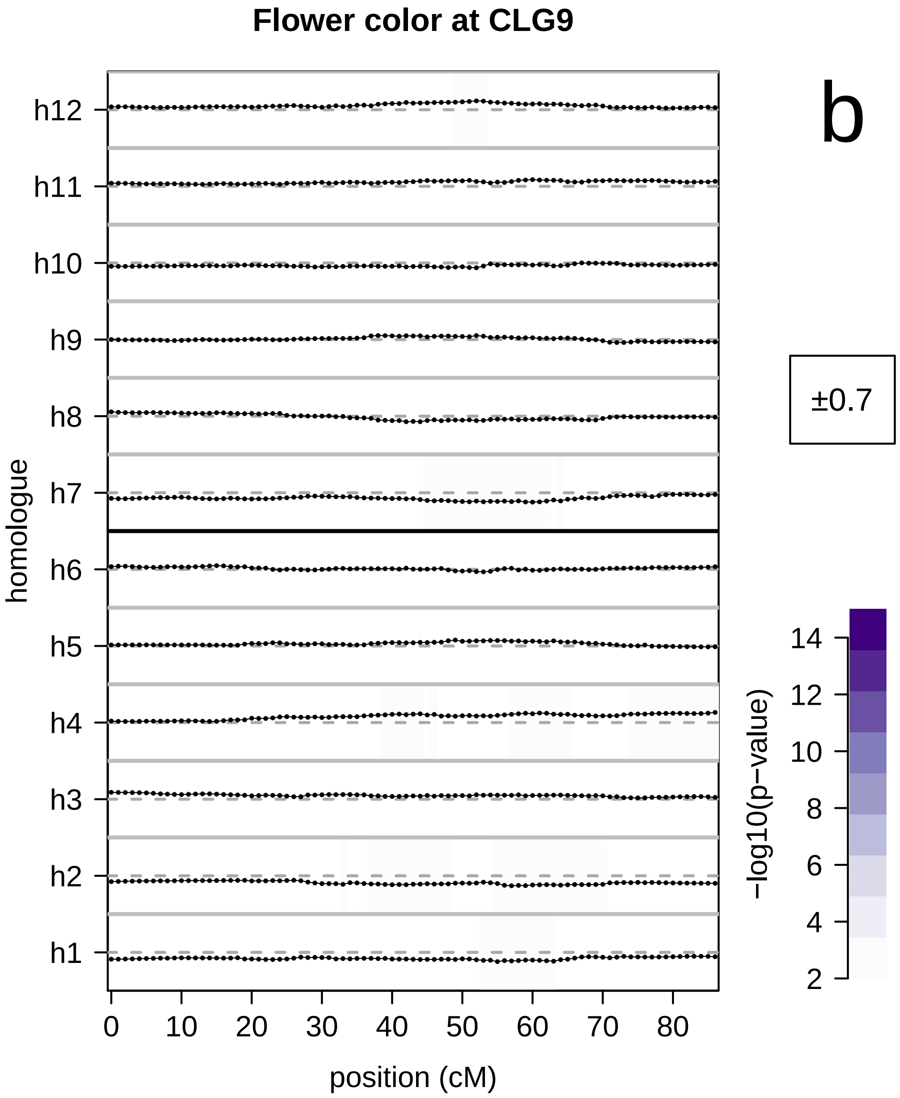
Flower color at CLG9
b
h12
h11
h10
h9
h8
h7
h6
h5
h4
h3
h2
h1
homologue
0
10
20
30
40
50
60
70
80
position (cM)
±0.7
−log10(p−value)
2
4
6
8
10
12
14

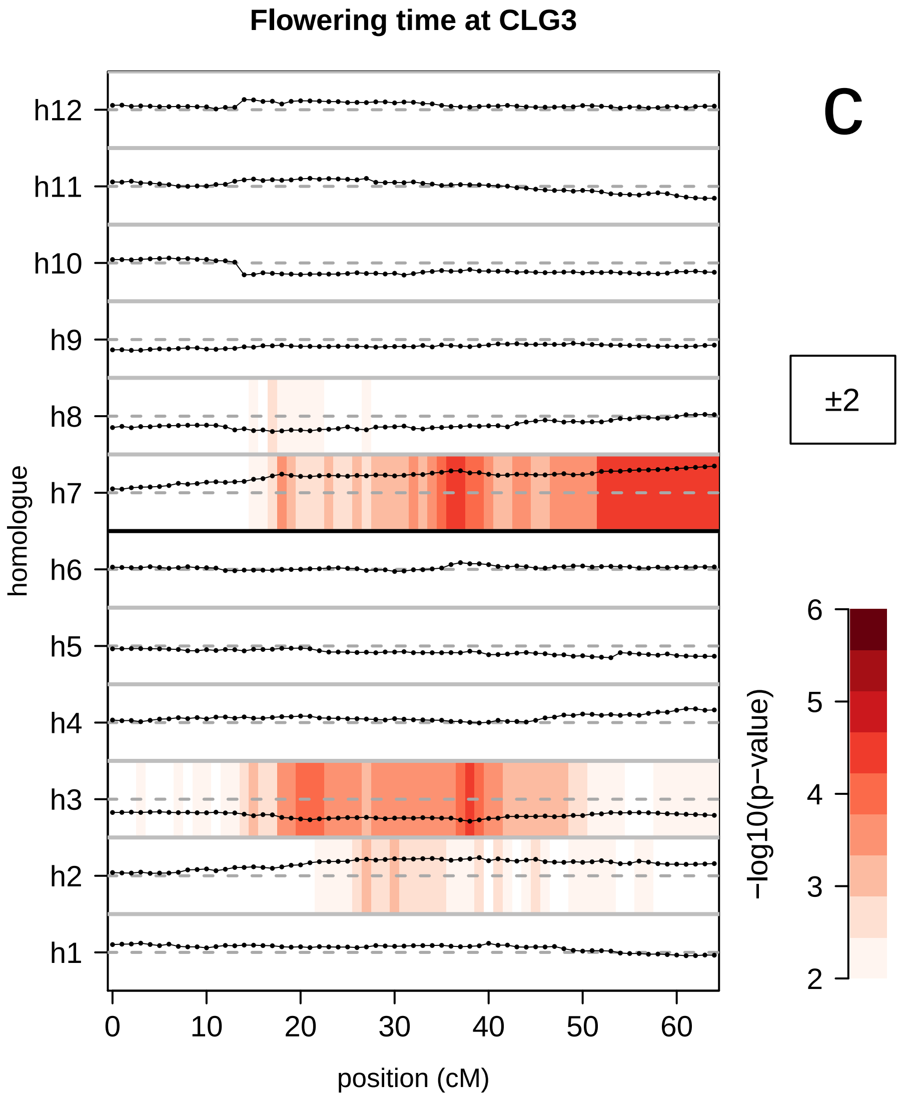

Flowering time at CLG3
C
h12
h11
h10
h9
h8
h7
h6
h5
h4
h3
h2
h1
homologue
±2
6
5
4
3
2
−log10(p−value)
0
10
20
30
40
50
60
position (cM)

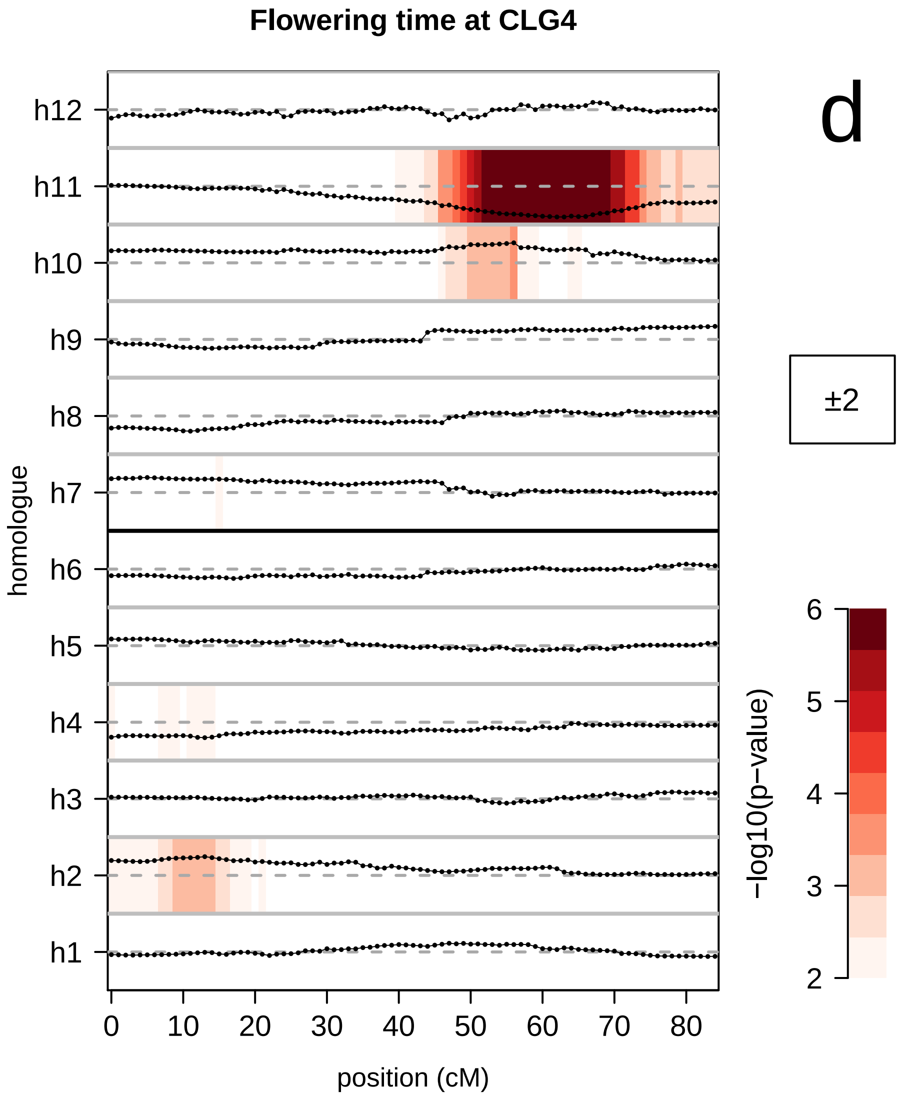
Flowering time at CLG4
d
h12
h11
h10
h9
h8
h7
h6
h5
h4
h3
h2
h1
homologue
0
10
20
30
40
50
60
70
80
position (cM)
±2
−log10(p−value)
6
5
4
3
2

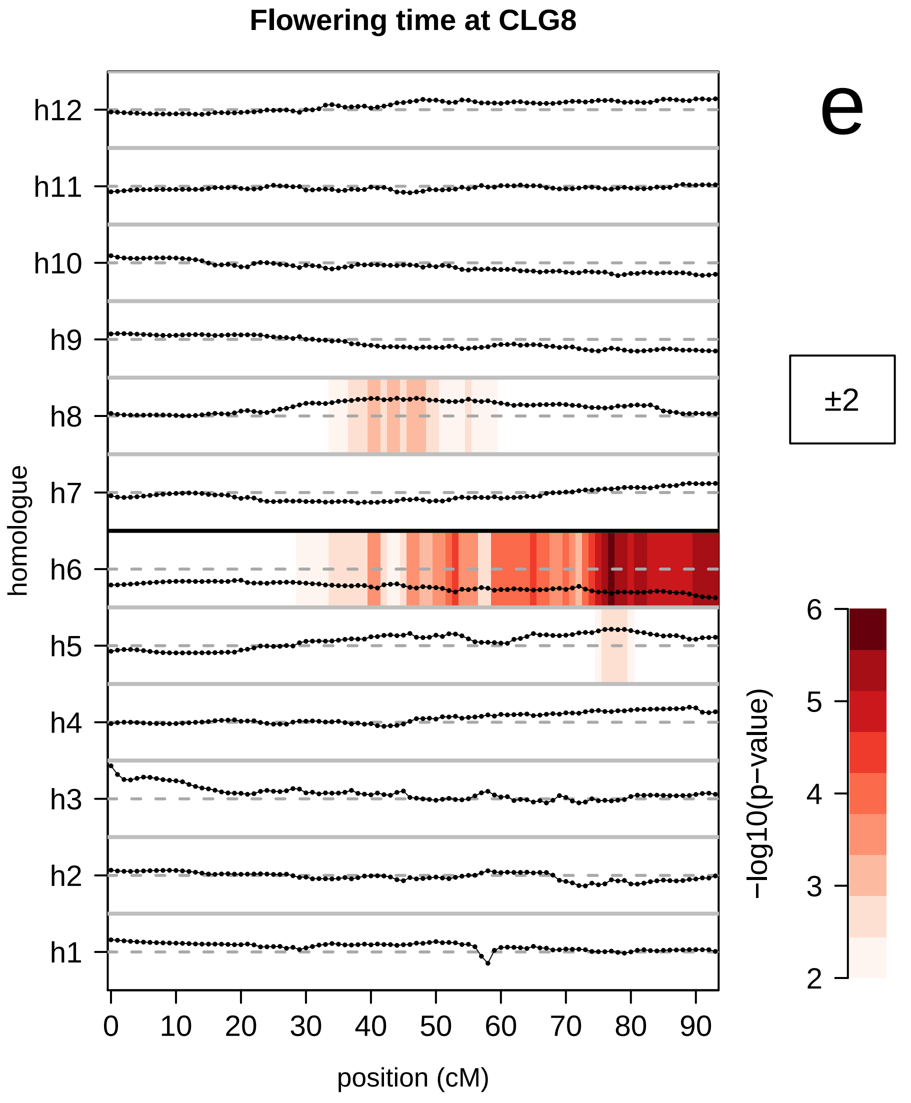

Flowering time at CLG8
e
±2
homologue
h12
h11
h10
h9
h8
h7
h6
h5
h4
h3
h2
h1
0
10
20
30
40
50
60
70
80
90
position (cM)
−log10(p−value)
2
3
4
5
6

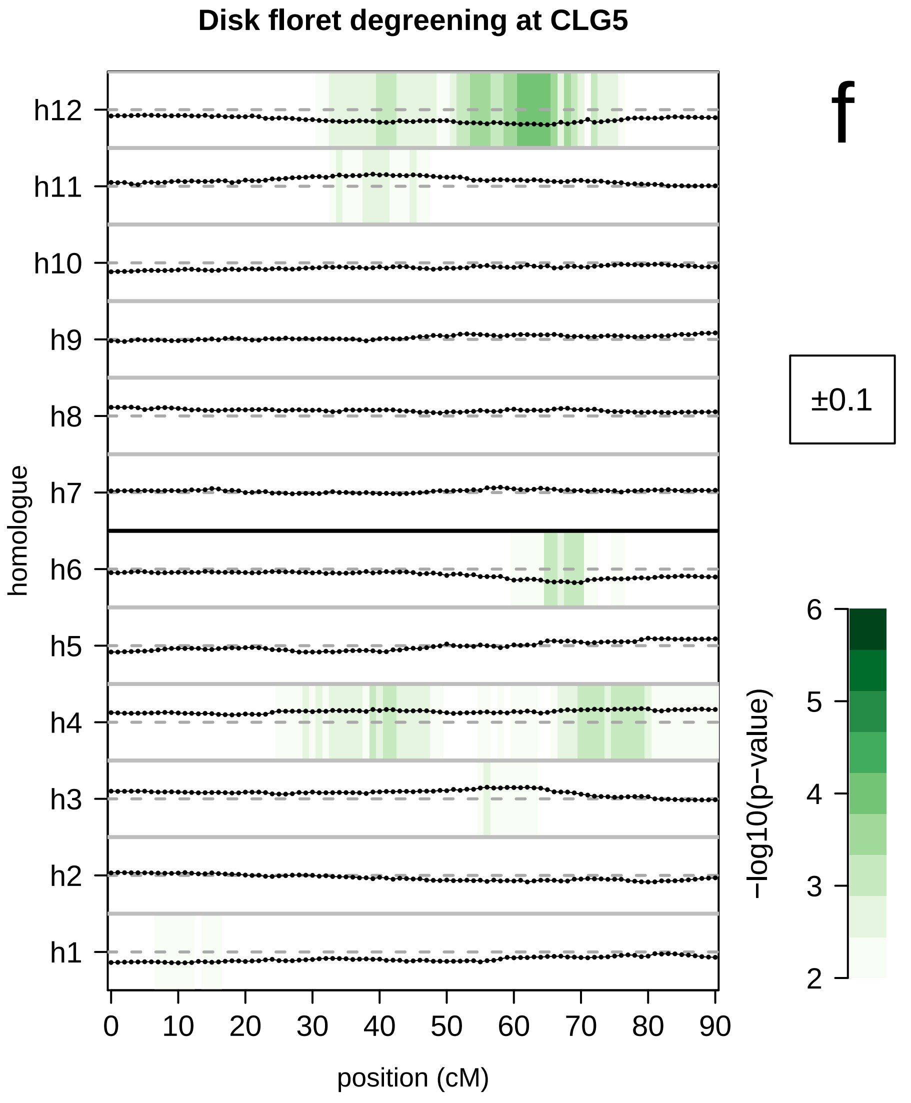
Disk floret degreening at CLG5
f
±0.1
h12
h11
h10
h9
h8
h7
h6
h5
h4
h3
h2
h1
homologue
0
10
20
30
40
50
60
70
80
90
position (cM)
−log10(p−value)
2
3
4
5
6

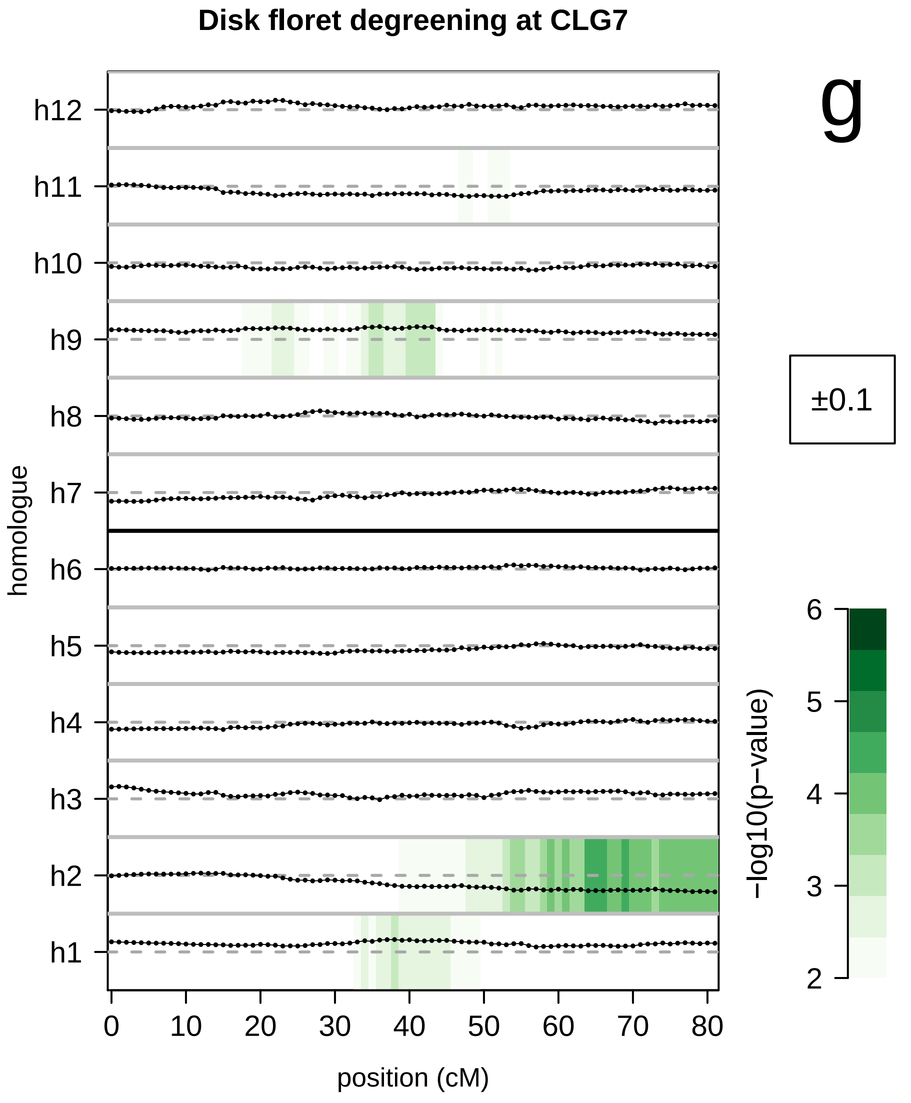

Disk floret degreening at CLG7
g
h12
h11
h10
h9
h8
h7
h6
h5
h4
h3
h2
h1
homologue
0
10
20
30
40
50
60
70
80
position (cM)
±0.1
−log10(p−value)
6
5
4
3
2

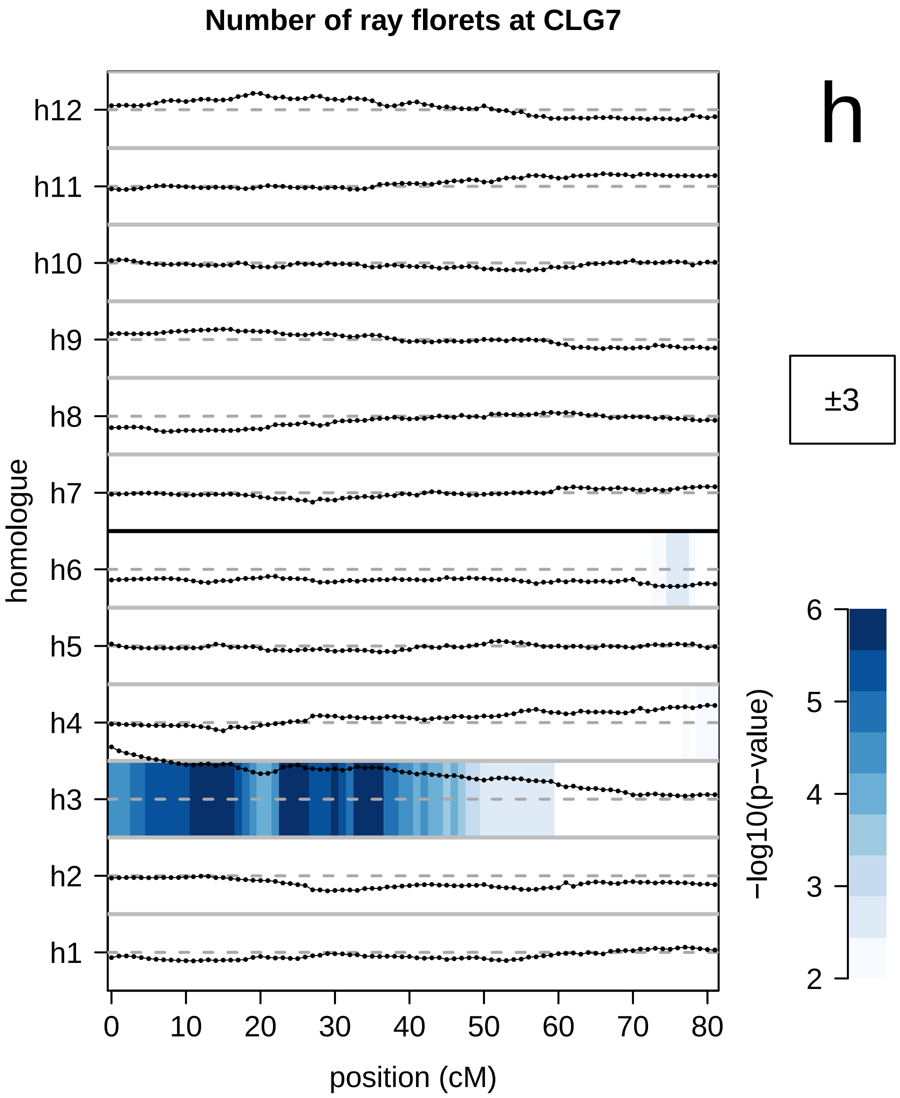
Number of ray florets at CLG7
h
h12
h11
h10
h9
h8
h7
h6
h5
h4
h3
h2
h1
homologue
0
10
20
30
40
50
60
70
80
position (cM)
±3
−log10(p−value)
6
5
4
3
2
